# HDAC Inhibition as Potential Therapeutic Strategy to Restore the Deregulated Immune Response in Severe COVID-19

**DOI:** 10.3389/fimmu.2022.841716

**Published:** 2022-05-03

**Authors:** Chiara Ripamonti, Valeria Spadotto, Pietro Pozzi, Andrea Stevenazzi, Barbara Vergani, Mattia Marchini, Giovanni Sandrone, Emanuele Bonetti, Luca Mazzarella, Saverio Minucci, Christian Steinkühler, Gianluca Fossati

**Affiliations:** ^1^ New Drug Incubator Department, Italfarmaco Group, Cinisello Balsamo, Italy; ^2^ Department of Experimental Oncology, IEO European Institute of Oncology IRCCS, Milan, Italy; ^3^ Department of Oncology and Hemato-oncology, University of Milan, Milan, Italy

**Keywords:** COVID-19, Histone deacetylases, inflammation, immune response, cytokines, T cell exhaustion

## Abstract

The COVID-19 pandemic has had a devastating impact worldwide and has been a great challenge for the scientific community. Vaccines against SARS-CoV-2 are now efficiently lessening COVID-19 mortality, although finding a cure for this infection is still a priority. An unbalanced immune response and the uncontrolled release of proinflammatory cytokines are features of COVID-19 pathophysiology and contribute to disease progression and worsening. Histone deacetylases (HDACs) have gained interest in immunology, as they regulate the innate and adaptative immune response at different levels. Inhibitors of these enzymes have already proven therapeutic potential in cancer and are currently being investigated for the treatment of autoimmune diseases. We thus tested the effects of different HDAC inhibitors, with a focus on a selective HDAC6 inhibitor, on immune and epithelial cells in *in vitro* models that mimic cells activation after viral infection. Our data indicate that HDAC inhibitors reduce cytokines release by airway epithelial cells, monocytes and macrophages. This anti-inflammatory effect occurs together with the reduction of monocytes activation and T cell exhaustion and with an increase of T cell differentiation towards a T central memory phenotype. Moreover, HDAC inhibitors hinder IFN-I expression and downstream effects in both airway epithelial cells and immune cells, thus potentially counteracting the negative effects promoted in critical COVID-19 patients by the late or persistent IFN-I pathway activation. All these data suggest that an epigenetic therapeutic approach based on HDAC inhibitors represents a promising pharmacological treatment for severe COVID-19 patients.

## Introduction

The coronavirus disease 2019 (COVID-19), caused by the severe acute respiratory syndrome coronavirus 2 (SARS-CoV-2), has rapidly turned into a pandemic. Although the majority of infected individuals are asymptomatic or develop mild symptoms, in about 20% of patients the infection causes a severe disease characterized by atypical interstitial bilateral pneumonia, acute respiratory distress syndrome (ARDS) and multi-organ dysfunction (1–4).

Airway epithelial cells of the upper and lower respiratory tracts play a fundamental role during the infection and are the main targets of the virus (5, 6). In fact, the airway epithelium is, on the one hand, the major site for the virus entry, thanks to the elevated expression of the SARS-CoV-2 receptor angiotensin-converting enzyme 2 (ACE2), while, on the other, it is fundamental to trigger the immune response against the invading virus (6–8). When SARS-CoV-2 enters its target cells, it triggers many signaling cascades including the activation of interferon regulatory factors (IRFs) and nuclear factor-kB (NF-κB) (9, 10). These transcription factors, in turn, enhance the activation of a powerful antiviral defense program mediated by the induction of type I and III interferons (IFN-I and IFN-III), interferon-stimulated genes (ISGs), cytokines and chemokines (11). The production and release of such pro-inflammatory signals, recalls effector cells and primes the adaptive immune response, which amplifies and sustains the initial response (9, 12). Thus, innate immunity has an essential role in recognizing and eliminating the virus, and there is increasing evidence that initial suboptimal and subsequent uncontrolled innate immune response during SARS-CoV-2 infection are defining features of COVID-19, determining disease severity and progression (10). In fact, innate immune response hyperactivation results in the excessive release of pro-inflammatory cytokines, including tumor necrosis factor-α (TNF-α), interleukin (IL)-1β, IL-2, IL-6, IFNα, IFNβ, IFNγ, inflammatory chemokines such as the CXC-chemokine ligand 10 (CXCL10) and monocyte chemoattractant proteins (CCL2, CCL3) (3, 12, 13). This systemic inflammatory response correlates with lung injury, ARDS and multiple organ failure (14–17). Several pieces of evidence indicate that an excessive alveolar monocyte/macrophage activation is associated with COVID-19 (1, 18, 19) and that the expansion of pro-inflammatory monocytes and monocyte-derived macrophages is evident in lungs of patients with severe disease when compared with those with moderate symptoms (20). Interestingly, COVID-19 shares several pathological features with sepsis (21, 22). Moreover, the development of sepsis due to bacterial superinfection occurred in COVID-19 patients admitted to intensive care units (23, 24). Therefore, considering certain aspects of COVID-19 as a sepsis-like disease it seems rational to develop therapies targeting the excessive host response with multiple approaches, taking into consideration the heterogeneous features of COVID-19 patients (22).

In parallel to the hyperactivation of innate immunity, COVID-19 patients show lymphopenia, with considerably reduced CD4^+^ and CD8^+^ T-cells (25–27). Numerous clinical studies have characterized changes in the number of CD8^+^ T-cells in mild and severe infections, underlying the importance of these cells in the progression of the disease. Peripheral CD8^+^ T-cells from COVID-19 patients display high levels of activation or exhaustion/senescence markers, linked to altered expression of lineage-specifying transcription factors and of chemokine receptors, with these alterations being more evident in patients with severe disease (26, 28).

Given the role of immune system hyperactivation in the pathogenesis of COVID-19, immunosuppressive treatments are essential for the clinical management of the disease, especially in severely ill patients. Corticosteroids are currently used for the treatment of severe COVID-19 patients since these drugs exert immunosuppressive and anti-inflammatory effects (29). Although recently several studies recommended the use of glucocorticoids in hospitalized patients requiring oxygen (30–32), the effectiveness of such drugs has for a long time been debated, also considering their side effects (30, 33, 34). The search for alternative anti-inflammatory therapies is therefore still necessary. Moreover, the presence of lymphopenia and exhausted circulating T-cells during COVID-19 suggests that more specific immune-therapies should be also considered.

All the pathological events described above depend on the alteration of many processes where epigenetic regulations have a key role. In particular, histone deacetylases (HDACs) have gained interest in immunology, as they were found to mediate the innate immune cell memory processes, as well as the tolerance towards endotoxins and T-cell differentiation and activation (35–38). In addition, HDAC3 and HDAC6 inhibition exerts anti-inflammatory activity and reduces pro-inflammatory cytokine production, improves clinical symptoms by blocking the production of IL-6 and TNF-α, and ameliorates clinical signs in models of systemic lupus erythematosus and colitis (39–43). Moreover, several studies suggested the use of HDACi to treat viral infections since acetylation and HDACs are involved at different levels during pathogens infections (44). Vorinostat, for instance, has been shown to have antiviral properties and to suppress adenovirus gene expression and replication (45, 46). Furthermore, growing evidence propose to consider HDACi as a potential therapeutic to COVID-19, given their anti-inflammatory and anti-thrombotic properties (47). HDACi also regulate SARS-CoV-2 entry in epithelial cells, both by downregulating ACE2 expression on the cell surface and by preventing ACE2-mediated entry of the virus (48, 49).

Currently, several pan-HDAC inhibitors (HDACi) are used in clinical trials and some are FDA-approved for the treatment of different types of cancer (50). However, clinical studies with pan-HDACi revealed multiple adverse events (51). To reduce side effects, inhibitors with increased selectivity for specific HDAC isoforms have been developed. In particular, in the last years highly selective inhibitors for HDAC6 have been discovered and several of them are now being evaluated in clinical trials (52). In fact, HDAC6 plays a key role as an immune checkpoint regulator in primary human melanoma cells (53) and the selective inhibition of HDAC6 isoform modulates Treg cells, enhancing their regulatory activity (54).

In light of these considerations, an epigenetic therapeutic approach based on HDAC inhibition could represent a promising new pharmacological treatment for COVID-19 patients. Therefore, we evaluated the effects of different HDACi on immune and epithelial cells in *in vitro* models that mimic Toll like Receptors (TLRs) activation after viral infection. In particular, we assessed and compared ITF3756, a new potent and selective HDAC6 inhibitor (52), with the pan inhibitors givinostat, a compound with orphan drug designation currently in phase 2 and 3 clinical trials for rare pathologies such as Duchenne and Becker muscular dystrophies (55) and polycythemia vera (56, 57), and the FDA-approved vorinostat. Given the fundamental role played by airway epithelial cells during SARS-CoV-2 infection, we tested the effects of HDAC inhibition in human primary epithelial cells from the upper and lower respiratory tracts. To simulate the epithelial cell response to viral-derived TLR activators, they were stimulated with the low molecular weight Poly I: C, a synthetic analogue of a double-stranded viral RNA (dsRNA) which binds the TLR3. We also tested HDACi on monocytes and macrophages stimulated with different TLR agonists, such as TLR7/8 and TLR4. Moreover, since the inflammation in COVID-19 patients is partially driven by NF-κB and characterized by increased TNF-α and IL-6 production (12), we investigated the global effect of the HDAC6 inhibitor ITF3756 on purified human monocytes stimulated with TNF-α. We also evaluated whether HDACi, and in particular ITF3756, modulate T-cells differentiation and exhaustion in an *in vitro* exhaustion model. Overall, our results indicate that HDAC inhibition has the potential to be used to reduce the morbidity associated with severe COVID-19 by acting on the innate and the adaptive immune system.

## Material and Methods

### Animal

C57BL6 female mice, 20-22 g, were obtained from Charles River Italy and kept in the animal facility for at least 5 days before use. Animals received food and water ad libitum and lighting was maintained on a 12h cycle. All *in vivo* studies were approved by the internal Animal Care and Use Committee in agreement with the Italian legislation D.Lgs 26/2014. The HDAC6 Knock-Out (HDAC6 KO) (58) colony was maintained and propagated for Italfarmaco by Charles River (Calco, Italy) under the license obtained from Friedrich Miescher Institute for Biomedical Research (Basel, Switzerland).

### Compounds and Reagents

LPS (TLR4 agonist) and R848 (Resiquimod, TLR7/8 agonist) were purchased from InvivoGen, human recombinat TNF-α was provided by Peprotech and anti-CD3/CD28 Dynabeads were supplied by Thermo Fisher Scientific. Dexamethasone was purchased from Sigma-Aldrich. Poly I:C Low Molecular weight (LMW) was purchased from InvivoGen. ITF3756, givinostat and vorinostat (SAHA) were synthetized by Italfarmaco Medicinal Chemistry Department and their *in vitro* activities on purified recombinant HDACs are shown in **Supplementary Figure 1**.

### Epithelial Cell Culture

Human Small Airway Epithelial Cells (SAEpC) (Promocell, C-12642) and Human Nasal Epithelial Cells (HNEpC) (Promocell, C-12620) were cultured in Small Airway Epithelial Cell Basal Medium (PromoCell, C-21270) and Airway Epithelial Cell Basal Medium (PromoCell, C-21060), respectively, supplemented with Growth medium kit (C-21170 and C- 21160, PromoCell) to obtain complete media. Cell cultures were maintained in a humidified tissue culture incubator at 37°C in 5% CO_2_. HNEpC (4.0x10^4^ cells in 96 well plates) and SAEpC (4.0x10^4^ cells in 96 well plates) were pre-treated with ITF3756, givinostat and vorinostat for 2 hours and then stimulated with Poly I: C 10μg/ml for 18h.

### Monocyte Purification and Activation

Peripheral blood mononuclear cells (PBMCs) used for the experiments were obtained from buffy coats of healthy donors separated over a Ficoll-Hypaque gradient (Biochrom). All samples tested negative for transmissible diseases as required for blood transfusion.

Monocytes were purified by positive selection from 100x10^6^ PBMC using CD14^+^ Isolation Kit (Miltenyi) and plated in RPMI supplemented with 10% Fetal Bovine Serum (FBS). Purified monocytes (1.0X10^6^/ml) were pre-treated for 2h with ITF3756 1 μM, givinostat 100 nM and vorinostat 1 μM in 12-well plates in 1-2 ml final volume. The cells were then stimulated with LPS (1 μg/ml) or R848 (5μg/ml) overnight (ON, approximately 18h). After incubation with HDACi and TLR stimuli, the cells were analyzed for the expression of CD40 and CD86 by flow cytometry (BD FACSVerse, BD Biosciences). Supernatants were collected and stored at -80°C for biomarker assays.

Purified monocytes, plated as above, were co-incubated with ITF3756 1 μM, givinostat 100 nM, vorinostat 1 μM, dexamethasone 100nM and R848 (5μg/ml) ON or were pre stimulated with R848 for 45 minutes and then treated with HDACi and dexamethasone ON. After incubation, cells were analyzed as indicated above.

For TNF-α stimulation, monocytes were purified by negative selection from 100x10^6^ PBMC using Pan Monocytes Isolation Kit (Miltenyi). Purified monocytes (1.0X10^6^/ml) were pre-treated for 2h with ITF3756 in 12-well plates in 1-2 ml final volume. The cells were then stimulated or not with TNF-α for 4h. After incubation with HDACi and TNF-α, the cells were collected, washed with PBS and rapidly frozen at -80C.

### Monocyte Differentiation Into Macrophages and Polarization

PBMC (3,0x10^6^/ml) were plated in 12 well-plates in RPMI medium supplemented with 0.1% FBS. After 3h, non-adherent cells were removed, and adherent cells (monocytes) were washed gently with PBS and incubated in 1ml of RPMI medium supplemented with 10% FBS (59, 60). Monocyte differentiation into unpolarized macrophages was induced for 7 days with M-CSF (50 ng/ml). Culture medium containing M-CSF was changed every 2-3 days. After 7 days, differentiated macrophages (M0) were treated with HDACi for 2h and then polarized ON with R848 (5μg/ml). Before treatment, cells were washed and stained with anti CD14 (Miltenyi) for 20min at room temperature (RT) and fluorescence was acquired using the flow cytometer BD FACSVerse. After incubation, the supernatats were collected and stored at -80°C for subsequent ELISA analysis. For 4 donors, cells were collected immediately frozen and stored at -80°C for subsequent qPCR analysis.

### ELISA

Cytokine detection (IL-1β, TNF-α and IL-6) was performed by ELISA (R&D System) according to Manufacturer’s instructions.

### LPS Induced Septic Shock *In Vivo*

C57BL/6 wildtype (WT) mice and C57BL/6 HDAC6 KO mice were challenged with a lethal dose of E.Coli LPS of 50 mg/kg by intraperitoneal (ip) injection. LPS (E.Coli 055: B5) was dissolved in saline and prepared at a concentration of 50mg/kg for a final volume of administration of 10mL/kg. The mice (7 per group) were monitored for 10 days and deaths were recorded to estimate the probability of surviving by Kaplan-Meier analysis.

C57BL/6 WT mice were challenged with lethal dose of E.Coli LPS 50 mg/kg ip. Animals were randomized and divided into two groups of 10 mice each. One group was treated with LPS only and the other with LPS followed by the HDAC6 inhibitor ITF3107 at 10mg/kg. ITF3107 was formulated in a PEG400 and H2O solution in a 1:1 ratio and DMSO at 5% final concentration. ITF3107 10 mg/kg was administrated once ip 3 hours after LPS treatment. The mice were monitored for 10 days and deaths were recorded to estimate the probability of surviving by Kaplan-Meier analysis.

### CD8^+^ T-Cells Purification, Activation and Phenotypic Analysis

CD8^+^ T cells were purified by positive selection from 100x10^6^ PBMC using CD8^+^ Isolation Kit (Miltenyi) and plated in RPMI medium supplemented with 10% FBS. 1.25x10^6^/ml CD8^+^ purified T-cells were pretreated for 2h with ITF3756 1μM, givinostat 100 nM and Vorinosat 1μM in 12-well plate. The cells were then stimulated with anti-CD3/CD28 Dynabeads (beads/CD8^+^ ratio 1:2) for 72h. At the end of this first round of stimulation, beads were removed using a magnet and cells were counted and washed. Subsequently, a second (48h) round of stimulations with fresh anti-CD3/CD28 beads in the presence or in the absence of HDACi was performed. In each stimulation step, the amount of beads used and the number of activated viable cells were kept proportionally equal.

At the end of the process of repetitive stimulation, a portion of cells were harvested and stained for surface exhaustion markers and phenotypic analysis (day5).

### Flow Cytometry

Purified monocytes were treated with FcR blocking reagent (Miltenyi) for 10 minutes at 4°C and stained with the following fluorochrome-conjugated antibodies: CD14 BB700 (BD Bioscience), CD40 PE (Miltenyi) and CD86 APC (BD Bioscience) for 20 minutes at RT.

Purified CD8^+^ T cells were stained with the following fluorochrome-conjugated antibodies: anti CD3 BB700 (clone SK7), anti CD8 APCH7 (clone SK1), anti CCR7 BV421 (clone 150503), anti CD45RA- PECy7 (clone H100), anti CD45RO FITC (clone UCHL1), anti CD62L APC (clone DREG-56), anti PD-1 APC (clone MIH4), anti LAG3 PE (clone T47-530), anti-TIM-3 BB700 (clone 344823) for 20 minutes at RT. All antibodies were purchased from BD Bioscience.

All samples were washed after antibody incubation and fluorescence was acquired using the flow cytometer BD FACSVerse. For CD8^+^ T-cells 1x10^4^ events were acquired, while for CD14^+^ cells 5x10^4^. Data were analyzed using BD FACSuit software.

### RNA Extraction and Reverse Transcription Quantitative PCR (qPCR)

For monocyte and macrophage gene expression analyses, total RNA was extracted with Trizol reagent (Thermo Fisher Scientific), following manufacturer’s instructions. RNA concentration and estimation of purity were determined by absorbance reading at 260 and 280 nm with NanoDrop ND-1000 Spectrophotometer (Thermo Scientific). cDNA was synthesized by reverse transcription using the Advantage RT-for-PCR Kit (Clontech) and used to perform SYBR Green based qPCR analysis. For airway epithelial cells gene expression analysis, cell lysis, total RNA extraction and cDNA synthesis were performed using the SYBR Green Fast Advanced Cells-to-Ct Kit (Thermo Fisher Scientific) following manufacturer’s protocol.

All the qPCR amplifications were achieved by Step One Plus or QuantStudio 12K Flex Real Time PCR systems (Thermo Fisher Scientific). The data were analyzed using the double delta Ct (DDCt) method and the 2^-DDCt values were displayed. The set of primers used are listed in **Table 1**.

**Table 1:** List of primers.

Gene	Product code	Supplier
18S	PPH05666E	SABioscience, Qiagen
BCL6	Hs.PT.56a.19673829g	IDT
BCL6	PPH00080E	SABioscience, Qiagen
CXCL10	PPH00765E	SABioscience, Qiagen
EOMES	Hs.PT.58.27752441	IDT
HAVCR2 (TIM-3)	Hs.PT.58.914695	IDT
HIF1-α	PPH01361B	SABioscience, Qiagen
IFIT1	PPH01332E	SABioscience, Qiagen
IFIT3	PPH02856A	SABioscience, Qiagen
IFIT5	PPH05801A	SABioscience, Qiagen
IFNα1	PPH01321A	SABioscience, Qiagen
IFNβ1	PPH00384E	SABioscience, Qiagen
IFNγ	PPH00380B	SABioscience, Qiagen
IL-1β	PPH00171B	SABioscience, Qiagen
IL6	Fwd: CACTGGCAGAAAACAACCTGAA Rev: ACCAGGCAAGTCTCCTCATTGA	Sigma Aldrich
IRF3	PPH02025A	SABioscience, Qiagen
IRF7	PPH02014E	SABioscience, Qiagen
LEF-1	Hs.PT.58.28328449	IDT
PDCD1 (PD-1)	PPH13086G	SABioscience, Qiagen
PRDM1(BLIMP1)	Hs.PT.56a.39313533g	IDT
RPL	PPH01020B	SABioscience, Qiagen
SLC2A1 (GLUT-1)	PPH02043C	SABioscience, Qiagen
TBX21(Tbe-t)	Hs.PT.58.3936407	IDT
TBX21	PPH00396A	SABioscience, Qiagen
TCF-7	Hs.PT.58.19831504	IDT
TNF-α	PPH00341E	SABioscience, Qiagen
TOX	Hs.PT.58.28002606	IDT

### RNA-Seq and Data Analysis

RNASeq of monocytes was performed by CRS4 (Cagliari, Italy). For TNF-α stimulated monocyte analysis, mRNA was isolated from 200 ng of total RNA using poly-T oligo-attached magnetic beads using two rounds of purification. Purified samples were processed using TruSeq RNA-Seq v2 Library Preparation Kit (Illumina) and pooled libraries were loaded on a Single End Flow Cell using the cBot System (Illumina) and the TruSeq PE Cluster Generation kit v3 (Illumina PE-401-3001). Sequencing was performed on a HiSeq2500 instrument (Illumina) using TruSeq SBS v3 reagents (Illumina FC-401-3001). Low quality ends and adapters were trimmed from single-end reads using TrimGalore (http://www.bioinformatics.babraham.ac.uk/projects/trim_galore/). Transcript abundance was estimated with Kallisto (61) and differentially expressed genes (DEGs) were identified using DeSeq2 R package and a FDR corrected p-value < 0.05. Hierarchical clustering analysis was performed using the gene expression values from DEGs. Specifically, Ward’s criterion for genes with 1 - (correlation coefficient) as a distance measure was used. Clustering heatmap was drawn using z-score across samples for each gene (62). Functional enrichment analysis was performed by the mean of the “enrichr” R package (EnrichR).

RNASeq of CD8_+_ T-cells was performed by Biogazelle (Zwijnaarde, Belgium). For CD3/CD28-stimulated CD8^+^ purified cells analysis, mRNA libraries were prepared using the TruSeq Stranded mRNA Library Prep Kit (Illumina), starting with 100ng RNA. Sequencing was performed on NextSeq 500 instrument according to the manufacturer’s instructions (Illumina). Reads were aligned to the reference genome using Bowtie2 (63) (v.2.2.3) and Annotation of the obtained sequences was performed using genome build GRCh38 and Ensembl 84. Spectral maps were generated using mpm R package based on DESeq2 normalized counts (DESeq2 R package (64), v1.8.2). Cumulative gene diversity was analyzed using QoRTs (65) (v1.0.7) based on DESeq2 normalized counts. Raw reads were normalized using the geometric mean-based method implemented in DESeq2 R package. DEGs were identified using DeSeq2 R package and a FDR corrected p-value < 0.05. Hierarchical clustering and functional enrichment analyses were performed as described above.

### Data Processing and Statistical Analysis

All data were analyzed using the GraphPad Prism 9 software. RM one-way ANOVA followed by Dunnett’s *post hoc* multiple comparison test was used for statistical analyses, otherwise differently specified. The assumption of normality was assessed graphically (i.e. Q-Q plots) and formally (i.e. Shapiro-Wilk test). The assumption of sphericity was assessed graphically (i.e. histograms) and/or formally (i.e. Mauchly’s test of sphericity). If the assumption of normality was violated the non-parametric Friedman test was used. If the assumption of sphericity was violated the Greenhouse–Geisser correction was used for RM one-way ANOVA. P-values ≤0.05 were considered statistically significant. In the graphs only the statistically significant differences are indicated. When not specified, the analysis gave a non-statistically significant difference.

## Results

### HDACi Treatment Downmodulates the Expression of Pro-Inflammatory Cytokines and IFN Pathway Genes in Epithelial Cells of the Upper and Lower Respiratory Tract

**Figure 1:**
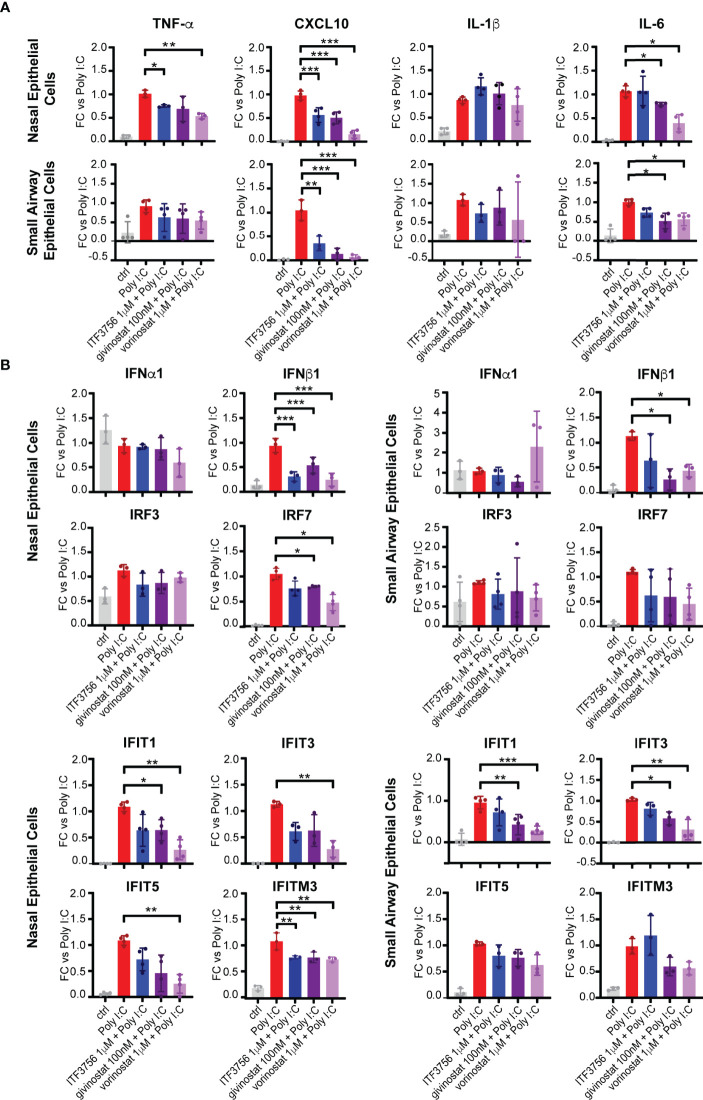
HDACi treatment downmodulates the expression of pro-inflammatory cytokines and IFN pathway genes in epithelial cells of the upper and lower respiratory tracts. Nasal epithelium primary cells (HNEpC) and lung epithelium primary cells (SAEpC) were pre-treated with ITF3756, givinostat and vorinostat for 2 hours and then stimulated with Poly I: C 10μg/ml for 18h. The expression of pro-inflammatory cytokines **(A)** and IFN pathway genes **(B)** was subsequently analyzed by qPCR. The graphs show the mean± SD of 3 or more biological replicates. FC = Fold changes. P values were calculated by RM one-way ANOVA as described in material and methods. *P < 0.05, ** < 0.01, *** < 0.001.

To mimic viral RNA recognition in airway epithelial cells, primary nasal and bronchial epithelial cells were stimulated with the TLR3 agonist Poly I:C in the presence of HDACi. Gene expression profiles of pro-inflammatory cytokines and of selected IFN pathway genes were evaluated. As expected, the expression of TNF-α, CXCL10, IL-1β and IL-6 was induced by Poly I:C stimulation in both nasal and pulmonary epithelial cells compared to the non-stimulated control **(** **Figure 1A** **)**. TNF-α, CXCL10 and IL-6 expression was reduced in both cell types in the presence of the pan inhibitors, but also after HDAC6 inhibition by ITF3756, except for IL-6 expression in nasal HNEpC. Instead, IL-1β was not modulated by the HDACi in either of the two cell types **(** **Figure 1A** **)**. IFNα1 expression was not induced by the stimulation with Poly I:C and none of the inhibitors modulated it in the airway epithelial cells. IFNβ1, on the contrary, was strongly expressed by cells after stimulation and its expression was reduced by all the HDACi in both cell types **(** **Figure 1B** **)**. HDAC inhibition did not modulate IRF3 expression, which was only slightly induced by TLR3 activation by Poly I:C in the nasal epithelial cells. However, here we observed a significant down-regulation of IRF7 expression **(** **Figure 1B** **)**. We next examined the expression of some ISGs as a read-out of the downstream effect of IFN signaling pathway activation, namely the members of the interferon-induced proteins with tetratricopeptide repeats (IFITs) family proteins, IFIT1, IFIT3, IFIT5 and the component of the IFN-induced transmembrane proteins (IFITMs), IFITM3. Both the HDAC6 selective and the pan inhibitors downregulated IFIT1, IFIT3, IFITM3 and, even if to a lower extent, also IFIT5, especially in the upper respiratory tract epithelial cells, confirming the general downregulation of the type I IFN signaling pathway by the tested inhibitors **(** **Figure 1B** **).** Interestingly, the angiotensin-converting enzyme 2 (ACE2), which act as a receptor for Sars-CoV-2 cell entry, is an ISG (8). Takahashi and colleagues showed that diverse HDAC inhibitors reduce ACE2 expression in gastric adenocarcinoma cell lines, and do not modulate the transmembrane serine protease 2 (TMPRSS2) (49), therefore, we investigated the possible effect of the HDACi on their expression in epithelial cells of the upper and lower respiratory tract. Stimulation of epithelial cells with Poly I:C induced a strong up-regulation of both ACE2 and TMPRSS2 **(** **Supplementary Figure 2** **).** In nasal epithelial cells, ACE2 expression was significantly reduced by both ITF3756 and pan HDACi, while in small airway epithelial cells its expression was only reduced by pan inhibitors and the results obtained with ITF3756 were rather variable **(** **Supplementary Figure 2** **)**. Conversely, TMPRSS2 expression was not regulated in either of the two cell types **(** **Supplementary Figure 2** **)**.

Together the results obtained indicate that HDAC6i and pan HDACi have an anti-inflammatory effect on epithelial cells from upper and lower respiratory tract and can, at least in part, downmodulate the IFN pathway as well.

### HDAC Inhibition Modulates *In Vitro* Immune Response in Stimulated Monocytes and Reduces *In Vivo* Mortality in Mice Subjected to LPS-Induced Septic Shock

Given the aggressive inflammatory response and the large production of pro-inflammatory cytokines occurring during severe COVID-19 infections, we investigated the potential anti-inflammatory effect of the HDAC6 inhibitor ITF3756 and of the 2 pan HDACi givinostat and vorinostat in monocytes stimulated with the TLR7/8 agonist R848. In addition, since during infections cell death and tissue damage favor TLR4 activation (66, 67), we also mimicked TLR4 activation in monocytes through LPS stimulation. We thus evaluated the expression of the costimulatory molecules CD40 and CD86, and the release of the pro-inflammatory cytokines TNF-α, IL-1β and IL-6.

**Figure 2:**
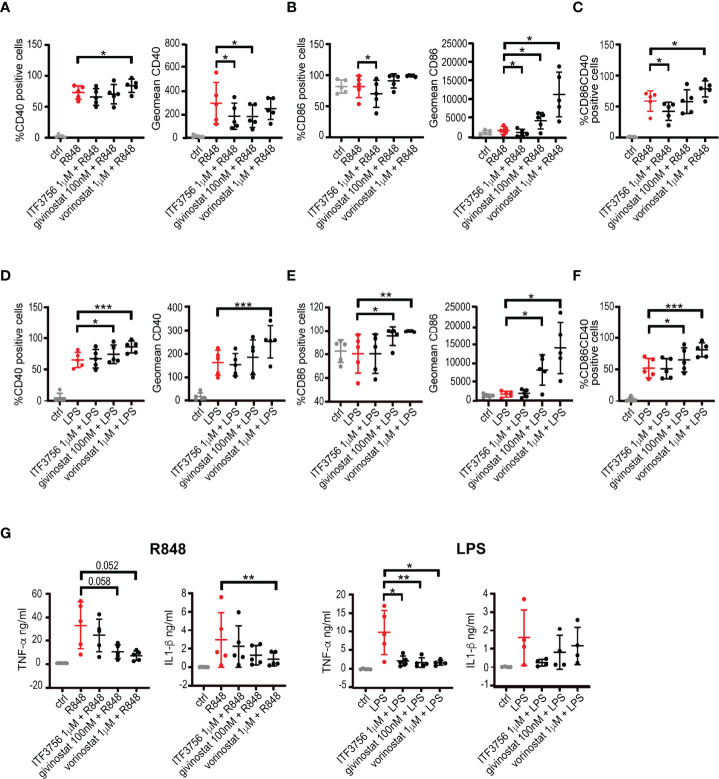
HDACi treatment modulates CD40 and CD86 surface markers expression and pro-inflammatory cytokines production in LPS or R848 stimulated monocytes. Human purified monocytes pre-treated with HDACi were stimulated with LPS (1μg/ml) or R848 (5μg/ml) and incubated ON. After incubation, cells were collected and stained with CD14, CD40 and CD86 antibodies and analysed by flow cytometry. Gate were performed on CD14^+^ population and dead cells were eliminated using Forward and Side scatters. Supernatants were collected and analysed for cytokines production. **(A–C)** Monocytes stimulated with R848: **(A)** percentage of positive cells and expression of CD40 **(B)** percentage of positive cells and expression of CD86 **(C)** CD40^+^/CD86^+^ double positive cells. **(D–F)** Monocytes stimulated with LPS: **(D)** percentage of positive cells and expression of CD40 **(E)** percentage of positive cells and expression of CD86 **(F)** CD40^+^/CD86^+^ double positive cells. **(G)** TNF-α and IL-1β production in R848 or LPS stimulated monocytes. Values on the graphs represent the mean ± SD obtained from 4 or more different donors analyzed in 3 separate experiments. P values were calculated by RM one-way ANOVA as described in material and methods for all samples. *P < 0.05, **< 0.01, ***< 0.001. Geomean is the geometrical mean fluorescence intensity.

**Figure 2** shows that R848 stimulation strongly upregulated CD40 surface expression and percentage of positive cells, while HDACi treatment with both ITF3756 and givinostat reduced CD40 expression significantly (**Figure 2A**). Conversely, R848 did not increase the percentage of CD86 positive cells and CD86 surface expression (**Figure 2B**). ITF3756 significantly downmodulated the percentage of CD86 positive cells and CD86 surface expression, whereas both pan HDACi significantly enhanced CD86 expression (**Figure 2B**). Given the effect on both surface molecules, R848 stimulation increased the percentage of CD40^+^/CD86^+^ double positive cells and the selective HDAC6 inhibitor significantly reduced this increment, while the pan HDACi vorinostat further increased the double positive population (**Figure 2C** and **Supplementary Figure 3**).

TLR4 activation is known to upregulate CD40 surface expression as we observed in our experimental model **(** **Figure 2D** **)**. After TLR4 activation, pan HDACi increased CD40 positive cells and expression and this increment became significant with vorinostat. All cells were positive for CD86 before stimulation, and LPS did not induce a further increase of CD86 expression **(** **Figure 2E** **)**. The selective HDAC6 inhibitor did not modulate CD86 expression, while both pan HDAC inhibitors significantly enhanced its expression **(** **Figure 2E** **)**. TLR4 activation increased the percentage of CD40^+^/CD86^+^ monocytes and this effect was further sustained in the presence of pan HDAC inhibitors, in particular vorinostat **(** **Figure 2F** **)**.

Stimulation of monocytes with R848 or LPS determined the release of inflammatory cytokines such as TNF-α, IL-1-β and IL-6 **(** **Figure 2G** and **Supplementary Figure 4** **)**. Pan HDAC inhibitors decreased TNF-α release induced by both stimuli, and reduced IL-1β secretion, although to a lower extent **(** **Figure 2G** **)**. Selective HDAC6 inhibition lead to a reduction of the pro-inflammatory cytokines TNF-α and IL-1β only when induced by TLR4 activation with LPS. None of the treatments was able to modulate IL-6 production from stimulated monocytes **(** **Supplementary Figure 4** **)**.

Pretreatment of monocytes with HDACi before stimulation with TLR agonists enable us to clearly identify the effect of the inhibitors on the inflammatory pathways and showed that the inhibitors could have a preventive anti-inflammatory effect. However, *in vivo*, cells in a tissue are subjected to waves of stimulations and, at the same moment, a proportion of cells can be stimulated by pro-inflammatory signals while another proportion is not. Thus, when administered *in vivo*, a drug can hit a cell before, during and after a stimulation. We thus performed experiments to better mimic what might occur *in vivo*. Monocytes were both co-incubated with HDACi and TLR7/8 agonist and pre-stimulated with R848 and then treated with the inhibitors. In addition, since dexamethasone is currently used in COVID-19 treatment, we used it as a positive control. **Supplementary Figure 5A** shows that ITF3756 decreases CD40 expression in monocytes with both co- and pre- stimulation with R848, in line with previous results. ITF3756 effect resulted the most comparable with dexamethasone, which clearly reduce CD40 expression in both models **(** **Supplementary Figure 5A** **)**. Of note, we observed an increase of CD40 expression after vorinostat exposure both with co- and pre-stimulation with R848, which we did not observe with pre-treatment. As for the experiments above, CD86 positive cells and CD86 expression were increased by pan HDACi in both models, while both ITF3756 and dexamethasone did not modulate CD86 **(** **Supplementary Figure 5B** **)**. The double positive population CD40+/CD86+ was slightly downmodulated only by dexamethasone both with co- and pre-stimulation with R848 **(** **Supplementary Figure 5C** **)**. Pan HDACi and dexamethasone clearly decreased TNF-α production both when co- and pre- stimulation was performed and in line with what was obtained with the pre-treatment model **(** **Supplementary Figure 5D** **)**. The effect on IL-1β secretion was confirmed for vorinostat and dexamethasone while givinostat was less effective **(** **Supplementary Figure 5E** **)**. Also, ITF3756 slightly modulated the production of both cytokines even if at a lower extent.

**Figure 3:**
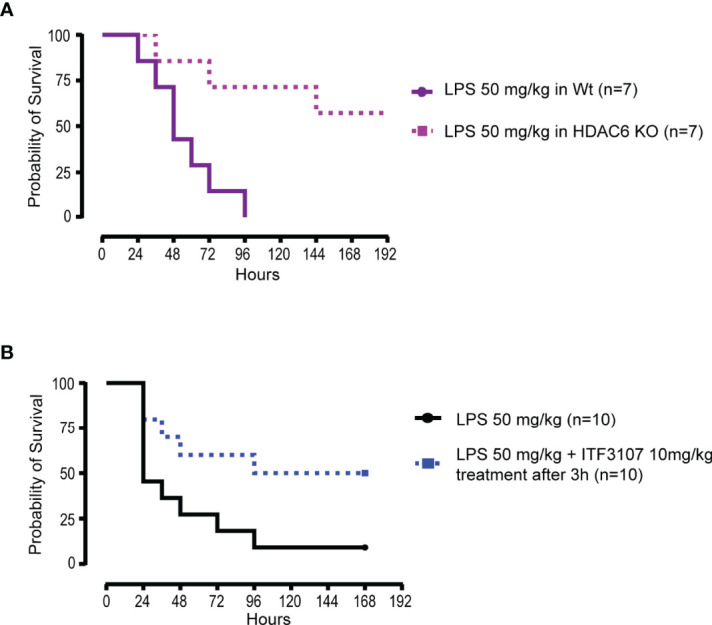
HDACi reduce mortality in mice subjected to LPS-induced septic shock *in vivo*. **(A)** C57BL/6 wildtype (WT) mice and C57BL/6 HDAC6 KnockOut (HDAC6 KO) mice were challenged with lethal dose of E.Coli LPS 50 mg/kg intraperitoneal (ip). The experiment was conducted for a maximum of 10 days and 7 mice for group were analyzed. The graph represents the survival curve of WT and HDAC6 KO mice treated with LPS. **(B)** C57BL/6 WT mice were challenged with lethal dose of E.Coli LPS 50 mg/kg ip. Before 3 hours from the LPS administration, animals were randomized and divided into two groups of 10 mice each. One group was treated with LPS and the other with the combination of LPS and ITF3107 (HDAC6 inhibitor) 10mg/kg. ITF3107 10 mg/kg was administrated once ip 3 hours after LPS treatment. The experimental design planned the animal treatment with HDAC6i with the septic shock already in progress. The experiment was conducted for a maximum of 10 days. The graph represents the survival curve of wild type mice treated with LPS and treated or not treated with ITF3107.

All together these results indicate that CD40 and CD86 expression and cytokine secretion are likely to be regulated by a complex interplay of acetylation processes. All three HDACi tested modulated both co-stimulatory surface markers and pro inflammatory cytokine release, even if to a different extent, based on their HDAC selectivity. It has been recognized that COVID-19 shares many pathophysiological and clinical features with sepsis. It has been extensively shown that pan HDACi dampen pro-inflammatory and innate immune responses in pre-clinical models of sepsis (68, 69). Interestingly, also HDAC6 is involved in sepsis and its inhibition improves survival in murine sepsis models (70). In agreement with this evidence, we observed that HDAC6 KO mice had a higher survival compared to wild type (WT) mice upon induction of septic shock by LPS **(** **Figure 3A** **)**. A similar effect on survival, was obtained in WT mice challenged with LPS and treated with another HDAC6 selective inhibitor, ITF3107, three hours after the LPS challenge **(** **Figure 3B** **)**. This HDAC6i is a precursor of ITF3756 and shared with it similar characteristics and selectivity for HDAC6 (52) **(** **Supplementary Figure 1** **)**.

All together these data show that HDAC inhibition modulates the activation and the inflammatory response induced in *in vitro* stimulated monocytes and reduces mice mortality in *in vivo* models of septic shock.

### The Selective HDAC6 Inhibitor ITF3756 Dampens the Proinflammatory Program Induced by TNF-α in Monocytes

**Figure 4:**
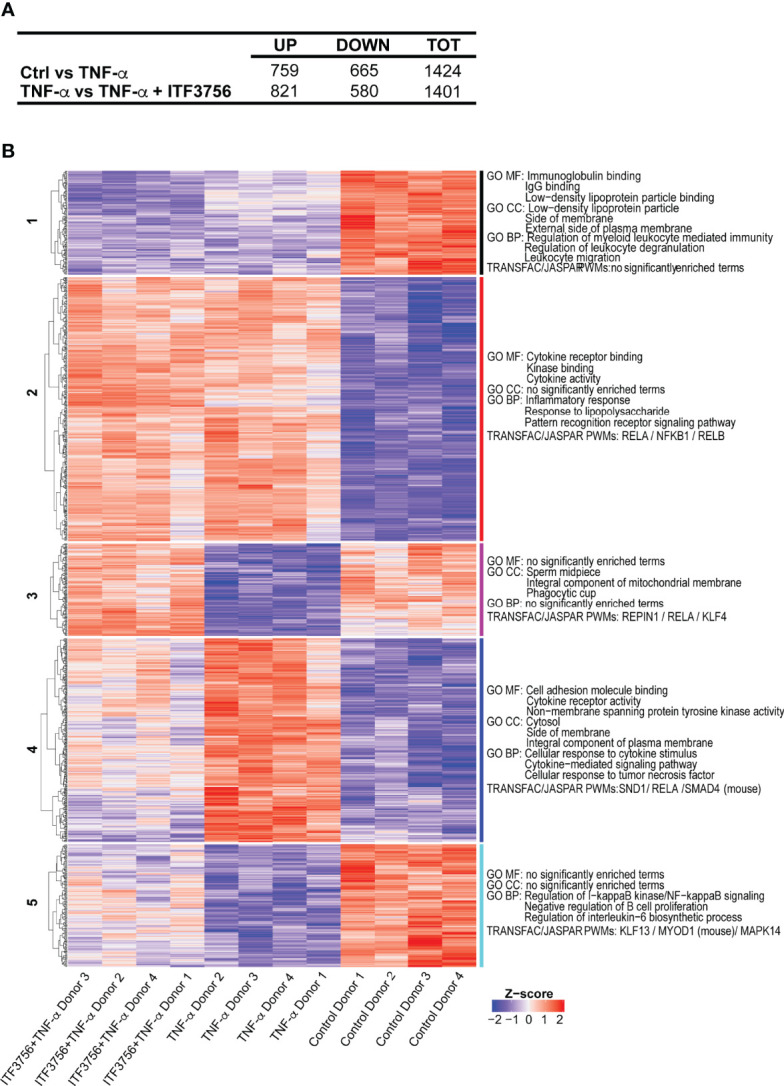
ITF3756 dampens the pro-inflammatory program induced by TNF-α in monocytes. Human monocytes pre-treated with ITF3756 1μM were stimulated with TNF-α (100ng/ml) for 4h. RNASeq analysis was performed on 4 different donors. **(A)** Summary of significantly differently expressed genes (DEGs). Up- and down- regulated genes were defined as follows: -1< log_2_(Fold change) <1 and padj<0.01. **(B)** The heatmap shows hierarchical clustering of RNASeq data of monocytes stimulated or not with TNF-α and treated with ITF3756. Gene Ontology of Molecular Function (MF), Biological Processes (BP) and Transcription Factor Binding motif enrichment (TRANFAC/JASPAR PWMs) is reported for each cluster. Top 3 gene sets per clusters are shown.

TNF-α has a relevant role in the inflammatory dysregulation during COVID-19 (25, 71). Since we found that HDAC6 inhibition seems effective in dampening the inflammatory response *in vitro* and plays a role in sepsis *in vivo*, we next focused on ITF3756 and asked whether the inhibitor could impinge on the TNF-α induced pro-inflammatory program in monocytes. We therefore purified human monocytes from peripheral blood mononuclear cells and stimulated them with TNF-α, in the presence or in the absence of ITF3756. Global gene expression analysis was performed by RNA sequencing. Monocyte stimulation with TNF-α caused a strong transcriptional response, with 759 and 665 differentially expressed (DEGs) up- and down-regulated genes, respectively **(** **Figure 4A** **)**. Hierarchical clustering analysis highlighted that ITF3756 counteracted the effect of TNF-α in activated monocytes, significantly modulating 1401 DEGs compared to TNF-α stimulated cells **(** **Figures 4A, B** and **Supplementary Table 1** **)**. Five transcriptional clusters were identified, and these could be separated in 2 sub-groups depending on the transcriptional effect of TNF-α stimulation: cluster 2 and 4 contain genes that were upregulated by TNF-α, while cluster 1, 3 and 5 include downregulated genes. Overall, ITF3756 antagonized the transcriptional effect of TNF-α stimulation, restoring the expression of genes close to that of the control, except for cluster 1 and partially for cluster 2, in which ITF3756 treatment downmodulated gene expression compared to TNF-α alone. Gene ontology (GO) and motif-finding analysis showed an enrichment of molecular functions (MF) and of biological processes (BP) related to cytokine activity and pathways, inflammatory response, Pattern Recognition Receptors and NF-kB signaling pathways in clusters 2 and 4 **(** **Figure 4** and **Supplementary Table 1** **)**. In fact, in these clusters, TNF-α stimulation enhanced the expression of genes related to inflammation and ITF3756 lessened (cluster 2) or reverted this effect (cluster 4). Cluster 3 and 5, which comprise genes that are downregulated by TNF-α stimulus, were also enriched for NF-kB binding motifs and for genes involved in the regulation of NF-kB signaling. Interestingly, in these two clusters, ITF3756 completely reverted (cluster 3) or attenuated (cluster 5) the TNF−α-induced downregulation. Thus, monocytes treated with TNF-α in the presence of ITF3756 show a gene expression profile similar to that of unstimulated control monocytes, which are in a resting condition and therefore have a minimal pro-inflammatory phenotype.

As reported in the literature, the cytokine storm occurring in COVID-19 patients leads to augmented expression of a number of pro-inflammatory genes in immune cells, besides cytokines and chemokines (71, 72). Several of these COVID-19 related genes were downregulated by ITF3756 treatment and therefore found in cluster 4 (**Supplementary Table 1**). Among them, PTX3 has been suggested to be a strong prognostic indicator of short-term mortality (73); augmented serum levels of the endothelial intracellular adhesion molecule-1 ICAM-1 are related to COVID-19 severity and indicate disruption of the endothelial barrier by SARS-CoV-2 (74, 75); PD-L1 is a protein related to dysfunctional myeloid cells in COVID-19 patients and, probably, the activation of the PD-L1-PD-1 axis causes CD8^+^ T-cells exhaustion (72); S100A12, is the gene encoding the EN-RAGE protein, that is expressed at high levels in blood myeloid cells in patients with severe COVID-19 (76); matrix metalloproteinase 9 (MMP-9) is an early indicator of respiratory failure in COVID-19 patients and an indicator of the role of extracellular matrix remodeling and fibrosis in this disorder (77). Altogether, these data indicate that ITF3756 modulates TNF-α signaling in *in vitro* stimulated monocytes, downregulating the expression of several inflammatory genes and of genes that have been described to be involved in COVID-19 and that play key roles in tissue degeneration occuring in seriously ill patients.

### HDACi Downregulate the Pro-Inflammatory Cytokine Production and Inhibit the IFN Pathway in TLR7/8-Stimulated Macrophages

**Figure 5:**
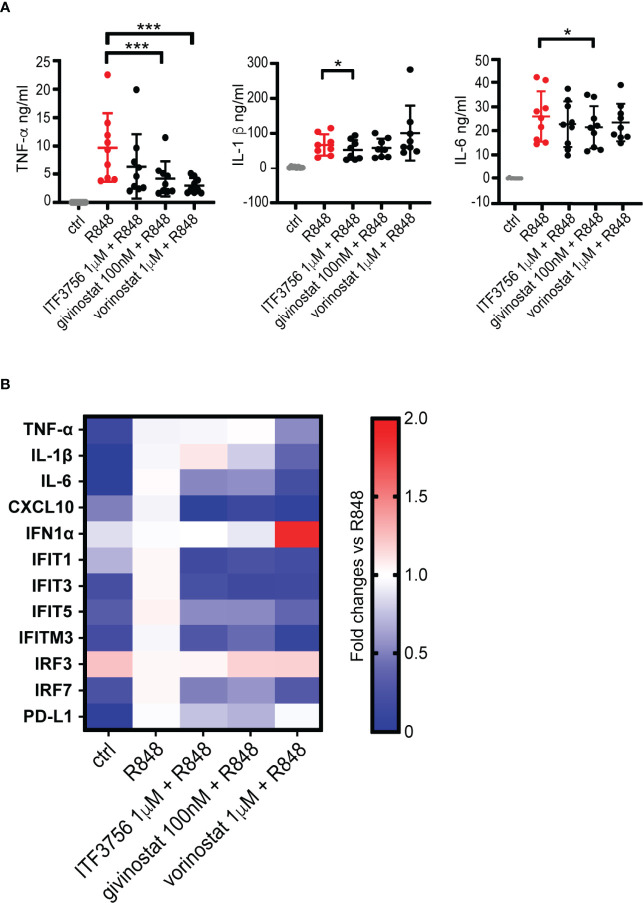
HDACi decrease pro-inflammatory cytokines production and gene expression in R848-stimulated macrophages. Human macrophages, differentiated from monocytes, were pre-treated with HDACi and stimulated with R848 (5μg/ml) ON. After incubation, supernatants were collected for ELISA analysis while cells were harvested for gene expression analysis by qPCR. **(A)** TNF-α, IL-1β and IL-6 ELISA. Values on the graphs represent the mean± SD values of at least 8 different donors. P values were calculated by RM one-way ANOVA followed as described in material and methods. ***p < 0.001. **(B)** Gene expression analysis by qPCR. Values on the graphs represent the mean± SD values obtained from 4 different donors.

A dysregulated macrophage activation state is one of the features of COVID-19 and stands at the basis of the hyper-inflammatory reaction that occurs during the disease (16, 20). Considering the results obtained on stimulated monocytes, we investigated whether cytokine release could be modulated by HDACi also after macrophage stimulation with the TLR7/8 agonist R848 *in vitro*. Human macrophages were thus differentiated from monocytes obtained from healthy donors, pretreated for 2h with HDAC6 and pan HDAC inhibitors and then stimulated with R848 for 18h. TNF-α, IL-1β and IL-6 were then measured in cell supernatants. The production of all the tested cytokines was strongly increased after macrophage stimulation **(** **Figure 5A** **)**. Both HDAC6i and pan HDACi downregulated the release of TNF-α when compared to the vehicle-treated control, while only ITF3756 significantly reduced IL-1β production. IL-6 was slightly reduced only by givinostat (**Figure 5A**). To extend the analysis of the effect of HDACi on macrophage activation, we also performed gene expression analysis of cytokines and IFN related genes. Consistently with cytokine measurement, we observed a slight reduction of the TNF-α and IL-1β expression by pan HDACi (**Figure 5B** and **Supplementary Figure 6**). CXCL10 was also strongly downmodulated by both pan and HDAC6 inhibitors. These results confirm that HDACi reduce pro-inflammatory cytokine expression and production (**Figure 5B** and **Supplementary Figure 6**).

We next assessed the expression of some IFN pathway genes which we found modulated by the HDACi in the previous experiments on monocytes. **Figure 5B** and **Supplementary Figure 6** show that R848 stimulation of macrophages did not induce IFNα expression compared to unstimulated cells, and that neither a 18h treatment with either pan HDACi or with ITF3756 affected IFNα expression, regardless of TLR 7/8 stimulation. Similarly, IRF3 expression was not modulated by HDAC inhibitors. However, IRF7 was strongly upregulated following R848 stimulation while it was significantly reduced by the action of both types of HDACi **(** **Figure 5B** and **Supplementary Figure 6** **)**. Both pan and HDAC6 selective inhibitors significantly downregulated the expression of IFIT1, IFIT3 and IFITM3, while IFIT5 was modulated only by ITF3756 **(** **Figure 5B** and **Supplementary Figure 6** **)**. Therefore, these data suggest that HDACi downregulate some of the key players of IFN signaling. The treatment of stimulated macrophages with HDACi also caused a reduction of PD-L1 expression in 3 out of 4 donors tested **(** **Figure 5B** and **Supplementary Figure 6** **)**. We also tested dexamethasone as positive control and investigated its effect on activated macrophages gene expression in 2 donors **(** **Supplementary Figure 6** **)**. In line with what observed in monocytes, dexamethasone showed a comparable effect with vorinostat in reducing the pro-inflammatory cytokines gene expression (TNF-α, IL-1β and IL-6). Conversely, dexamethasone did not modulate the IFN pathway genes (IFIT1, IFIT3 and IFITM3) compared to the HDACi.

Overall, the data shown here indicate that HDACi, both HDAC6 selective and pan, downregulate the TLR7/8 pathway leading to diminished pro-inflammatory cytokine production and to the inhibition of the IFN pathway in macrophages.

### HDAC Inhibition Reprograms *In Vitro* CD8^+^ T-Cell Development Toward Central Memory and Less Exhausted Phenotype

During COVID-19, T-cells counts are significantly reduced in patients with a severe disease and the surviving T-cells appear functionally exhausted and with a phenotype closely related to T-effector cells (26). Reverting the exhaustion process or reducing the activation status of T-cells in a dysregulated highly inflammatory environment, such as the one occurring in COVID-19, could be crucial to maintain a more prolonged and effective response against the virus and, at same time, to reduce the inflammatory process. Thus, we investigated whether HDAC6i and pan HDACi affect T-cell differentiation and the expression of exhaustion markers during an exhaustion process *in vitro*. To recapitulate the T-cell exhaustion process (78), purified CD8^+^ T-cells were repeatedly stimulated (day 0, 3) with anti CD3/CD28 coated beads in the presence or in the absence of the inhibitors. The ability of HDACi to alter the exhaustion and effector-memory differentiation process was evaluated at day 5 of the exhaustion process.

**Figure 6:**
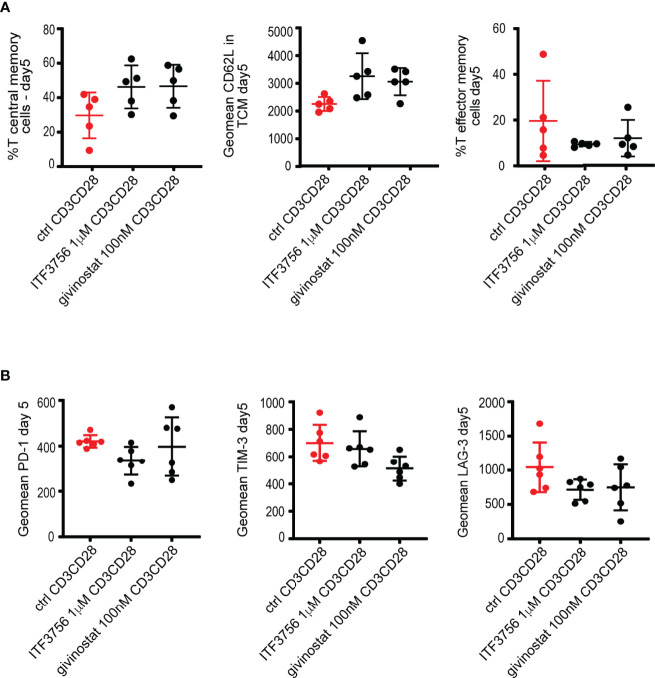
HDACi treatment promotes T central memory phenotype and decreases T exhaustion markers in CD3CD28-stimulated CD8^+^ purified cells. Purified CD8^+^ cells were pre-treated with HDAC6i or Pan HDACi for 2 hours and then stimulated with anti-CD3/CD28 beads for 3 days followed by another run of stimulation in the presence of the inhibitors for 2 days. Cells were counted at the end of each stimulation period, beads removed, and cells placed in new medium with fresh beads for the next stimulation. Characterization of exhaustion markers and T cells phenotype was performed by flow cytometry. **(A)** T central memory (TCM) positive cells (CD45RO^+^CCR7^+^CD62L^+^), CD62L expression in TCM and T Effector memory cells (TEM) (CD45RO^+^CCR7^-^CD62L^-^ cells). **(B)** T exhaustion markers PD-1, TIM-3 and LAG-3 as geomean fluorescence intensity. Values on the graphs represent the mean± SD values of 3 experiments carried out on a total of at least 5 different donors. P values were calculated by RM one-way as described in material and methods. Geomean is the geometrical mean fluorescence intensity.

We compared the effect of ITF3756 with givinostat, on human purified CD8^+^ T-cells collected from 5 to 6 different donors. **Figure 6A** shows that both ITF3756 and givinostat increased T-central memory (TCM) cells (defined as CD45RO^+^CCR7^+^CD62L^+^) and CD62L expression, a marker of naïve/memory T cells, in TCM cells while they reduced T-effector memory cells. Exhausted T-cells typically express co-inhibitory receptors such as PD-1, TIM-3 and LAG-3. The pan HDACi reduced the expression of TIM-3 and LAG-3 but not that of PD-1 while the selective HDAC6 inhibition was effective in reducing LAG-3 and PD-1 but was not sufficient to reduce TIM-3 expression **(** **Figure 6B** **)**.

Vorinostat was also tested on CD8^+^ T-cells, however we found that it was cytotoxic when used at the same concentration used with all the previous experiments in the other cell types (1μM). The reduction of its concentration to 100 nM resulted non-toxic and the overall effect was comparable to that of givinostat in two donors (data not shown).

Collectively our results indicate that HDACs inhibition promotes the increase of a TCM population and the reduction of key T-cell exhaustion markers. Of note, a low dose of givinostat, and the selective HDAC6i show similar effects. It is worth noting that givinostat is also a potent inhibitor of HDAC6, suggesting that this subtype plays a key role in the process studied.

### The Selective HDAC6i ITF3756 Changes the Landscape of CD8^+^ T-Cell Gene Expression During *In Vitro* Development

From the experiments described above, HDAC6i controls the *in vitro* CD8^+^ T-cell fate upon repeated TCR stimulation. Thinking of a possible therapeutic application of HDAC inhibitors in COVID-19, we next concentrated the further analyses on ITF3756 to better assess the potential of selective inhibitors on T-cell differentiation and exhaustion. In fact, both givinostat and vorinostat, as other pan-HDACi, are less tolerated compared with HDAC6 selective inhibitors, which show a better safety profile.

**Figure 7:**
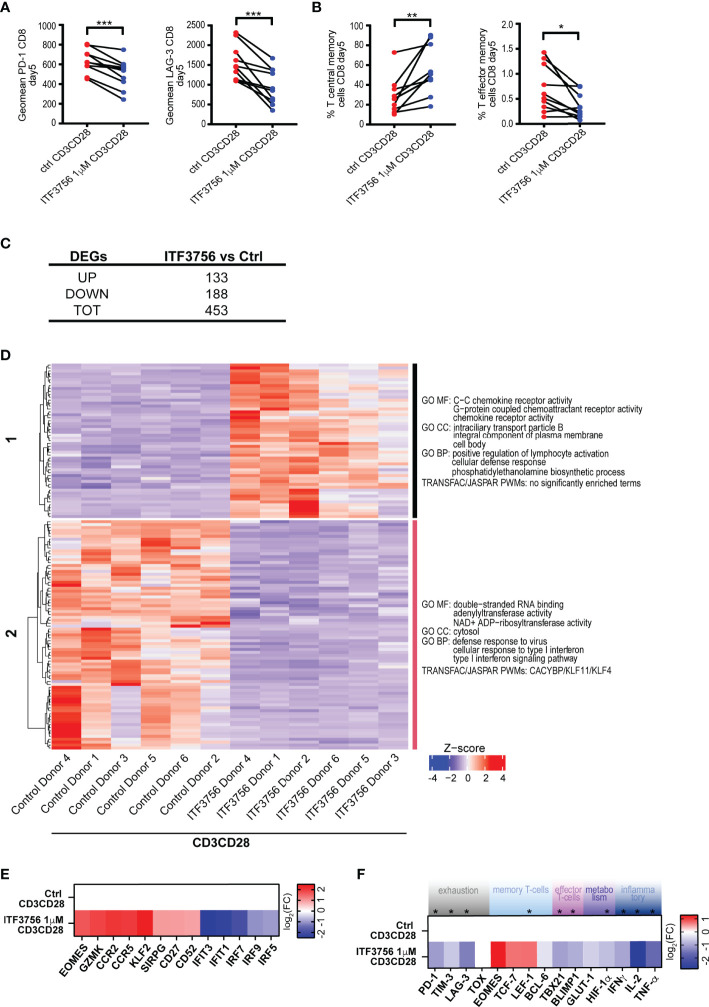
ITF3756 treatment reduced exhaustion and increased T central memory phenotype in CD3/CD28-stimulated CD8^+^ purified T-cells during the exhaustion process. Purified CD8^+^ T-cells were pre-treated with ITF3756 for 2 hours and then stimulated with anti-CD3/CD28 beads for 3 days followed by another run of stimulation in the presence of the inhibitor for 2 days. Cells were counted at the end of each stimulation period, beads removed, and cells placed in new medium with fresh beads for the next stimulation. At day 5 after stimulation cells were collected and analysed by flow cytometry, qPCR and RNASeq. **(A, B)** Characterization of exhaustion markers and T-cells phenotype was performed by flow cytometry. **(A)** Exhaustion markers **(B)** T central memory phenotype. Paired t-test analysis of 10 donors at day 5 of exhaustion process is shown *P≤ 0.05, **≤ 0.01, ***< 0.001. Geomean is the geometrical mean fluorescence intensity. **(C–F)** Gene expression analysis of mRNA extracted at day 5 by RNASeq and by qPCR in 6 donors. **(C)** Summary of significantly differently expressed genes (DEGs). Up- and down- regulated genes were defined as follows: -1< log_2_(Fold change) <1 and padj<0.01. **(D)** The heatmap shows hierarchical clustering of RNASeq data of CD3/CD28-stimulated CD8^+^ T-cells treated or not with ITF3756. Gene Ontology of Molecular Function (MF), Biological Processes (BP) and Transcription Factor Binding motif enrichment (TRANFAC/JASPAR PWMs) is reported for each cluster. Top 3 gene sets per clusters are shown. **(E)** The heatmap shows the modulation of specific genes observed in the RNASeq analysis. Log_2_ Fold Changes of ITF3756 modulated genes compared to the untreated control were reported. **(F)** Real Time qPCR analysis of gene expression of a selected panel of genes known to be involved in T-cells exhaustion, activation and differentiation in 6 donors. The heatmap shows log_2_ Fold Changes of ITF3756 modulated genes compared to the untreated control. P values were calculated by Paired t-test analysis. Asterisks indicate statistically significant changes (P ≤ 0.05).

We therefore focused on ITF3756 and performed an expanded and thorough analysis of the effect of the inhibitor on the T-cell population and exhaustion on a larger number of donors. Paired analysis of 10 donors is shown in **Figures 7A, B**. As expected, ITF3756 treatment significantly upregulated T central memory cells and significantly downmodulated both T-effector memory cells and the expression of the exhaustion markers PD-1 and LAG-3 **(** **Figures 7A, B** **)**. During the process of repeated TCR stimulation, T-cell differentiation is driven by epigenetic activation or repression of specific genes. To understand how ITF3756 may change the landscape of CD8^+^ T-cell gene expression, we performed RNASeq analysis on T-cells isolated from the 6 donors that better responded to the treatment. 453 significantly regulated DEGs were identified after ITF3756 exposure, of which 133 up-regulated and 188 down-regulated compared to untreated CD8^+^ T-cells **(** **Figure 7C** **)**. Hierarchical clustering analysis clearly separated genes in two clusters in which ITF3756 treatment completely reverted gene expression compared to the control (**Figure 7D** and **Supplementary Table 2**). GO analysis showed an enrichment of molecular functions and of biological processes related to chemokine receptor activity, positive regulation of lymphocyte activation and cellular defense response pathways in clusters 1, while in cluster 2 emerged an enrichment for cellular response to type I interferon and its signaling pathway (**Figure 7D** and **Supplementary Table 2**). To better investigate the characteristics of these cells after ITF3756 treatment, we looked at specific genes in each cluster and found the up-regulation of several genes related to the T-cell memory phenotypes (**Figure 7E** and **Supplementary Table 2**). Among them Granzyme K, that together with granzyme A was reported to be expressed in an early memory stage of CD8^+^ T-cells development (79), the chemokine receptor genes CCR2 and CCR5, CD27 (80), CD52 (81), SIRPG (82) and KLF2 (Kruppel-like factor 2) zinc finger transcription factor expressed on CD8^+^ T –cells, especially in naïve and memory T-cells, and involved in T-cells trafficking (83). We also confirmed that ITF3756 treatment showed a reduction of the IFN pathway genes: IRF5, IRF7 and IRF9 were downmodulated and, consequently, also the ISGs IFIT1 and IFIT3. These data agree with those obtained in TLR7/8 stimulated macrophages and TNF-α stimulated monocytes, in which HDAC inhibition caused a strong reduction of IFN pathway and ISG.

We next further assessed the expression of other genes related to T-cell differentiation and exhaustion after ITF3756 treatment by Real Time PCR, on the same samples analyzed by RNASeq **(** **Figure 7F** **)**. ITF3756 reduced the expression of PD-1, TIM-3 and LAG-3. It also increased EOMES, TCF-7 and LEF-1, that are related to T-cell memory phenotypes, while it slightly decreased the effector memory markers, TBX21 and BLIMP1. The expression of BCL6, involved in the inhibition of T cell exhaustion, and TOX, characteristically expressed in exhausted cells, were not clearly modulated. Conversely, inflammatory and proliferation cytokine genes (IFNγ, TNF-α and IL-2) were down-modulated, as well as the metabolism genes HIF-1α and GLUT-1.

Overall, we showed that HDAC6 inhibition by ITF3756 modulates T-cells by reducing their exhaustion and maintaining them in a more memory-like phenotype, as documented both at the gene and protein levels. T-cells with these characteristics could promote a more sustained response in COVID-19 patients.

## Discussion

Acetylation is a post-translational modification orchestrating several cell functions at different levels, from signal transduction to epigenetic gene regulation. Its fine balance is altered in non-homeostatic conditions, therefore pharmacologic modulation of protein acetylation has great potential for the treatment of a number of pathologies. In COVID-19 patients, a dysregulated response of both innate and adaptive immune system is the main driver of the development of the most severe forms. Currently, many clinical trials with HDAC inhibitors with different selectivity are ongoing, especially for oncological diseases. Still, we believe that the anti-inflammatory properties of HDAC inhibitors should also be exploited. Givinostat, for example, is currently in clinical trials for Duchenne muscular dystrophy and exhibits antifibrotic and anti-inflammatory effects on muscle tissue (55). In this study, we assessed the immunomodulatory effect of three different HDACi in *in vitro* models that mimic the activation and the immune response induced by viruses in different types of cells. We used givinostat and vorinostat at two different concentrations, both clinically relevant (84, 85), based on previous in-house experiments where the activity of vorinostat was 5 to 10-fold lower compared to givinostat. Interestingly, at these two different concentrations, the two pan HDACi had an opposite effect on the regulation of CD86 expression in monocytes and vorinostat resulted toxic on CD8^+^cells while it was not on the other cells used.

In particular, we showed for the first time that both pan and HDAC6 selective inhibitors, have an anti-inflammatory effect on primary epithelial cells from the upper and lower respiratory tracts stimulated with a TLR3 agonist, reducing the expression of TNF-α, CXCL10 and IL-6. Moreover, we observed a clear downregulation of the type I IFN pathway in these cells. In TLR signaling, IRF3 and IRF7 are positive regulators of type I IFNs and while IRF3 is costitutively expressed, IRF7 is normally expressed at low levels in most cells (86, 87). The data presented here show that, in airway epithelial cells, IRF7, IFNβ and all the ISGs tested were significantly downmodulated by HDACi, clearly indicating a dampening of the pathway. Accordingly, we also showed that both HDAC6 selective and pan inhibitors downregulate the expression of the ISG ACE2 in airway epithelial cells. HDACs are well known regulators of both IFN and TLR signaling (88). Previous reports described the role of HDACs in both the production of type I IFN or the response to type I IFN through the regulation of ISGs (88–92). Moreover, they can act both as positive and negative regulators of TLR signaling, depending on the specific enzyme isoform and on the context (88). Interestingly, high levels of type I IFN is a negative prognostic marker in COVID-19 patients (71). In fact, in SARS-CoV-2 infections, the virus delays the activation of the IFN responses, which are induced too late and often when patients have already developed a severe disease (93, 94). Thus, while therapies based on the administration of IFNs may be beneficial in the early phases of the pathology, therapies that maintain IFNs at low levels may be protective at late stages and when the disease is already severe.

Resident lung macrophages and monocytes are reported to be the main source of cytokine storm and inflammation in severe COVID-19 cases (1). We assessed the effect of HDACi on circulating monocytes stimulated with a TLR7/8 agonist to mimic *in vitro* the innate immune response triggered by the recognition of SARS-CoV-2 genetic material. Since COVID-19 patients admitted to intensive care units frequently present bacterial superinfections with massive TLR4 stimulation, and because cell death and tissue damage during the infection favors TLR4 activation (66, 67), we also mimicked TLR4 activation in monocytes through LPS stimulation. Overall, HDACi modulated markers of monocytes activation and reduced cytokines release when cells were stimulated with the TLR agonists used. ITF3756 downregulates CD40 and CD86 markers when TLR7/8 were activated, while it did not reduce TNF-α and IL-1β release. Conversely, it was effective in reducing cytokine release and CD40 expression in LPS-stimulated monocytes. ITF3756 induced downregulation of the co-stimulatory receptors CD40 and CD86 after TLR7/8 stimulation could still be relevant in reducing inflammation even in the absence of cytokine reduction. In fact, it is reported the CD40-/- mice are resistant to LPS-induced lung injury and polymicrobial sepsis (95–98). Pan HDACi, instead, reduced the production of cytokines in monocytes stimulated with both stimuli, but induced the expression of CD86, in particular upon vorinostat treatment. Nevertheless, pan HDAC treated monocytes showed a less inflammatory phenotype with likely enhanced antigen presenting cells capability, both of which could be useful characteristics in reducing inflammation in COVID-19 (99). These results highlight how the differential inhibition of HDAC isoforms can influence the cellular response compared to pan-HDAC inhibition.

These results were obtained by a pre-incubation of the cells with the inhibitor. This model mainly mimics a prophylactic approach although *in vivo* the target cells will experience all the three main possible scenarios when they encounter the drug: a) the cells have already been stimulated; b) the cells are stimulated at the same time of drug contact; c) the cells are stimulated after the drug contact. When we tested different HDACi treatment schedules, in which the anti-inflammatory therapy was administered both together and after the onset of the inflammatory program, HDACi confirmed their anti-inflammatory properties on activated monocytes. In particular, vorinostat confirmed the data obtained by pre-treating the cells, namely clear induction of CD86 expression and strong inhibition of cytokine production. Givinostat showed a milder effect compared to vorinostat, probably because of the lower dose used. Also ITF3756 confirmed its tendency to reduce CD40 expression but it did not affect CD86 when the HDAC inhibition was performed together or after the inflammation onset. Reduction of cytokines by pan HDACi was confirmed both in the co- and pre-stimulation models. Interestingly, we observed an overall reduction of cytokine release by ITF3756 compared with previous experiments, in which the results obtained among different donors were more variable. Dexamethasone is currently used to treat COVID-19, we therefore tested it in both co- and pre- stimulation models, where it caused similar effects on co-stimulatory markers and on cytokine production. It robustly reduced surface inflammatory receptors and cytokine released, likely maintaining monocytes in a more inactivated state compared to HDACi. Overall, the effect of HDACi on co-stimulatory molecules on activated monocytes when the inhibitors were used together and after the inflammation onset, resulted milder than when they were given before the inflammatory stimulus. This might indicate that they need a longer exposure time to efficiently modulate activated cells.

The data obtained *in vitro* on the reduction of the inflammatory response in TLRs-stimulated monocytes were further sustained and expanded by *in vivo* experiments. HDAC6 genetic ablation reduced the mortality in mice after LPS-induced septic shock. Also pharmacological administration of an HDAC6 inhibitor given three hours after LPS challenge could significantly reduce the mortality. These experiments confirmed that HDAC6 plays a key role in sepsis and, in agreement with our results, literature data reported that HDAC6i ameliorate sepsis-induced ARDS by regulating both innate and adaptive immunity, preserving endothelial barrier function and attenuating pro-inflammatory gene transcription (70, 100, 101).

In addition, we found that ITF3756 acts on two different levels: on one side, it reduces the release of TNF-α by stimulated monocytes, and on the other, it modulates the pro-inflammatory program elicited by TNF-α itself in these cells. In fact, global analysis of gene expression of monocytes stimulated with TNF-α and treated with ITF3756 showed a downregulation of pro-inflammatory pathways, in particular of the NF-kB inflammatory response pathways. Moreover, in TNF-α activated monocytes, ITF3756 reduces the expression of additional biomarkers of severe COVID-19, such as PTX3, ICAM-1, S100A12, PD-L1 and MMP9, further supporting a potential role for this molecule in the treatment of the disease. Interestingly, among these genes, PD-L1 is highly expressed in basophils, eosinophils, monocytes and NK cells of severe COVID-19 patients (72). Chen and colleagues suggest that the cytokine storm may be responsible for the increase of PD-L1 expression (102) and, in fact, it has been demonstrated that PD-L1 expression is regulated by various signaling pathways which are triggered by IL-6 and TNF-α (103). Moreover, the PD-L1/PD-1 axis is a major regulator of CD8^+^ T-cell function and the PD-L1-PD-1 interaction could determine CD8^+^ T-cell dysfunction and apoptosis (103). Thus, PD-L1 expression represents another important target that ITF3756 may regulate at two levels, directly acting on PD-L1 transcription upon TNF-α stimulation in monocytes and reducing the release of TNF-α by the same cells. Another interesting gene that we found downregulated by ITF3756 in TNF-α stimulated monocytes is MMP9. MMP9 promotes the degradation of the alveolar capillary barrier during acute lung injury, further stimulating migration of inflammatory cells and destruction of lung tissue (104). Even if the primary source of MMP9 are neutrophils the strong reduction of gene expression observed in monocytes suggests that ITF3756 could have a similar effect in other cell types, with a possibly positive effect in SARS-CoV-2 patients.

Finally, consistently with what was observed in monocytes and airway epithelial cells, HDACi, and in particular ITF3756, reduced cytokine release and downregulated the expression of pro-inflammatory and IFN-related genes also in macrophages stimulated with the TLR7/8 agonist. Of note, PD-L1 downregulation by HDACi was observed also in stimulated macrophages.

The comparison of HDACi with dexamethasone, further support a possible therapeutic epigenetic approach for COVID-19. In fact, HDACi showed a comparable reduction of cytokine expression on monocytes and macrophages. However, differently from dexamethasone, HDACi downregulate the IFN-pathway genes, which could be particularly useful in the context of severe COVID-19.

Our data support the notion that HDACi have also a role in regulating the adaptive immune response, which is dysregulated in severe COVID-19 patients as well. Both pan and HDAC6 selective inhibitors were able to reduce the T-cell exhaustion phenotype, as indicated by the downregulation of PD-1 and LAG-3, both at the transcript and protein level, and by the downmodulation of TIM-3 at the gene expression level. Moreover, treatments with HDACi induced the CD8^+^cell differentiation towards a memory phenotype. These CD8^+^ memory cells, in fact, exhibit a central memory phenotype with modulation of both surface markers and many genes determining this phenotype. Among them, CCR5 and CCR2 are upregulated by ITF3756. Surface expression of chemokine receptors is associated with human T-cell subsets with different migratory capacity, effector function and cytokine production (86). Thus, based on the different activation and differentiation state, early, intermediate and late TCM cells differently express chemokine receptors and change the extent of specific cytokine production. Our data suggest that ITF3756 drives CD8^+^cells to an intermediate TCM phase, in which CCR2, CCR5 and CCR7 are up-regulated and cytokine expression is reduced (105–107). Interestingly, the accelerated recruitment of antigen-specific memory CD8^+^ T-cells to the lung airways during respiratory virus infections is dependent on CCR5 expression (108). Moreover, as we observed in airway epithelial cells and in myeloid cells, reduction of type I IFN target genes is obtained also in T-cells upon ITF3756 treatment. This reduction, as previously discussed, may be beneficial for critical COVID-19 patients, since in the late stages of the disease IFN-I may exacerbate inflammation and suppress the adaptive immune response by inducing T-cell exhaustion (109).

**Figure 8:**
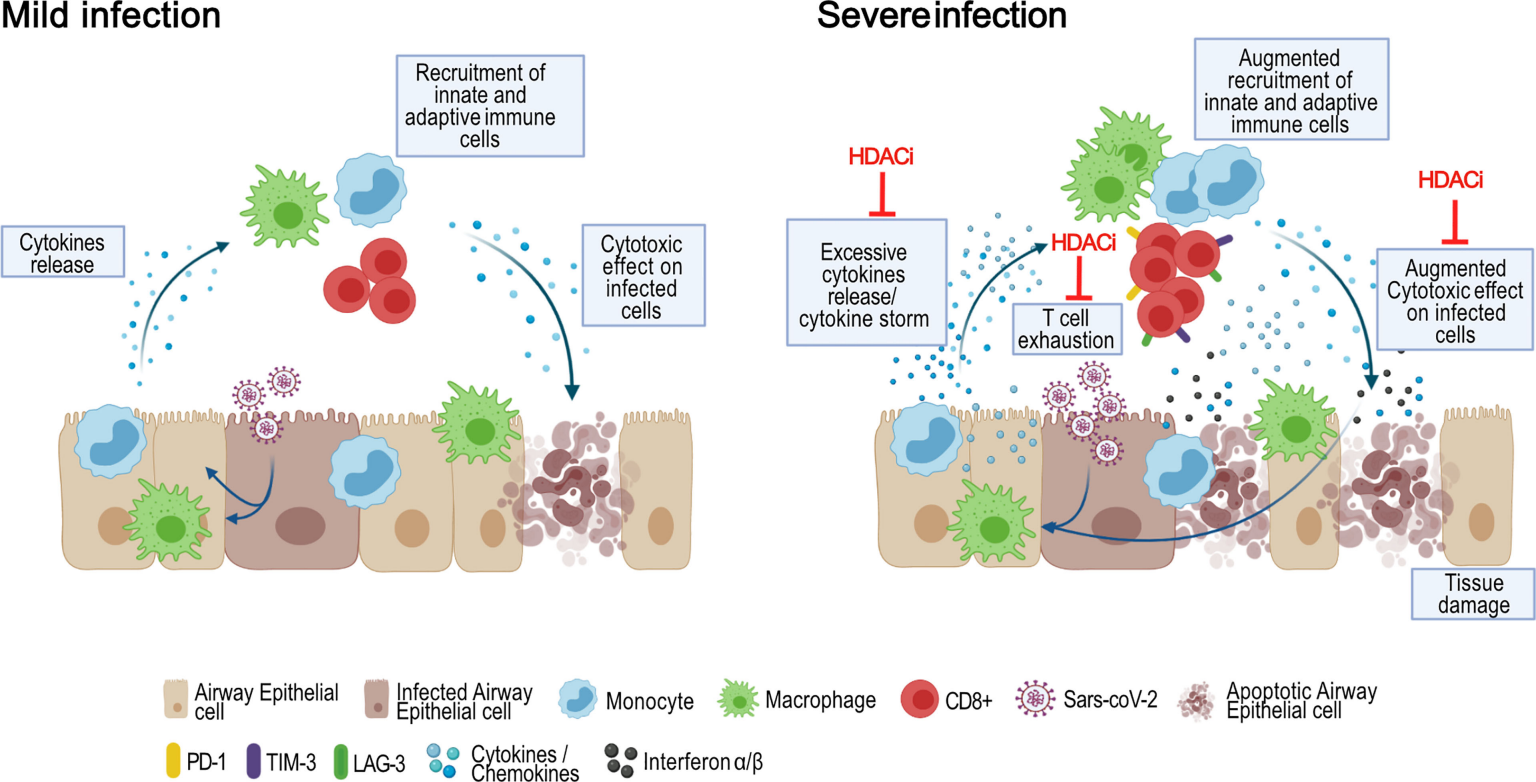
Schematic overview of the pathogenesis and outcomes of COVID-19 and potential therapeutic effects of HDACi. In mild Sars-CoV-2 infection cases, infected airway epithelial cells recall and activate monocytes and resident macrophages through the release of cytokines and chemokines. These cells, in turn, trigger a specific innate immune response to eliminate the pathogen. The increase in pro-inflammatory signals foster the recruitment of adaptive immune cells, including CD4^+^ and CD8^+^ T cells. This immune response leads to the elimination of the infected cells, viral clearance and resolution of the disease. In severe infections instead, a dysfunctional immune response characterized by an excessive infiltration of monocytes and macrophages, lymphopenia and increased T cell exhaustion, lead to unaffected viral elimination and lung tissue damage. Moreover, the hyperproduced cytokines circulate to other organs, causing multi-organ damage. In this context, HDACi act at multiple levels: i) reducing cytokines release by epithelial cells, monocytes and macrophages; ii) inhibiting the pro-inflammatory effects of IFN-I iii) reducing monocyte activation and T cell exhaustion and promoting T cell differentiation versus a T central memory phenotype. Created with BioRender.com.

As summarized in **Figure 8**, HDACi may act at multiple levels during severe SARS-CoV-2 infection. First, they may reduce cytokine release by airway epithelial cells, monocytes and macrophages, limiting COVID-19 hyperinflammation. Second, they may counteract the negative effects promoted in critically ill patients by the late or persistent IFN-I pathway activation, since they inhibit IFN-I expression and downstream effects in both airway epithelial cells and the immune cells. Third, HDACi reduce monocyte activation and T-cell exhaustion and promote T-cell differentiation towards a T central memory phenotype.

Overall, the data presented here support the notion of testing HDACi, and possibly HDAC6 selective inhibitors for severe COVID-19 treatment. Given the results obtained in this study, we envisage a possible application for HDACi in the late stage of the of COVID-19. This concept is also supported by recent evidence indicating that the HDAC6i Ricolinostat is effective in dampening the overproduction of neutrophil extracellular trap formation, which is characteristic of several pathologies including COVID-19 (110). Of note, the inhibitors we tested are in advanced stage of development. Givinostat is in phase III clinical trial and ITF3756 has successfully completed the toxicological studies and it is going to enter a phase 1 clinical trial soon while vorinostat is an FDA approved drug.

Targeting hyperinflammation is a promising therapeutic strategy against COVID-19. Currently, many clinical trials are testing the ability of different drugs to reduce inflammation during SARS-CoV-2 infections. Among these are monoclonal antibodies (mAb) against various cytokines and small molecule inhibitors targeting chemokines and their receptors, such as IL-6, CCR2 and CCR5 and TNF-α signaling (1, 111). However, hitting a single target may limit hyperinflammation but it is unlikely to resolve the whole immune dysregulation affecting COVID-19 patients. In this view, HDACi may have a high therapeutic potential, restoring crucial dysregulated aspects of innate and adaptive immunity.

## Data Availability Statement

The complete datasets regarding RNASeq presented in this article are not readily available because they are the object of a patent application that we will submit shortly. Therefore, we need to preserve the intellectual properties of our Company. Requests to access the datasets should be directed to the corresponding Author at the following email address: g.fossati@italfarmaco.com.

## Ethics Statement

The animal study was reviewed and approved by Internal Animal Care and Use Committee in agreement with the Italian legislation D.Lgs 26/2014.

## Author Contributions

GF, CS, and CR conceived the project idea. VS and CR performed the experiments, analyzed the data, and wrote the manuscript. EB contributed to the bioinformatic analysis. PP performed the *in vivo* experiments and analyzed the data. AS, BV, MM, and GS designed and synthesized ITF3756. GF, CS, LM, and SM contributed to the critical reading and revision of the manuscript. All authors read and approved the final manuscript.

## Funding

This work was supported by Regione Lombardia (POR FESR 2014-2020, ID 1827871”EPICO”).

## Conflict of Interest

CR, VS, PP, AS, BV, MM, GS, GF, and CS are employees of Italfarmaco.

The remaining authors declare that the research was conducted in the absence of any commercial or financial relationships that could be construed as a potential conflict of interest.

## Publisher’s Note

All claims expressed in this article are solely those of the authors and do not necessarily represent those of their affiliated organizations, or those of the publisher, the editors and the reviewers. Any product that may be evaluated in this article, or claim that may be made by its manufacturer, is not guaranteed or endorsed by the publisher.
