# Supplementary material for: HDAC Inhibition as Potential Therapeutic Strategy to Restore the Deregulated Immune Response in Severe COVID-19
Source: Front Immunol. 2022 May 3;13:841716. doi: 10.3389/fimmu.2022.841716 (PMC9111747; doi:10.3389/fimmu.2022.841716)
Supplement: Supplementary file 1 [file Presentation_1.pptx]

## Slide 1
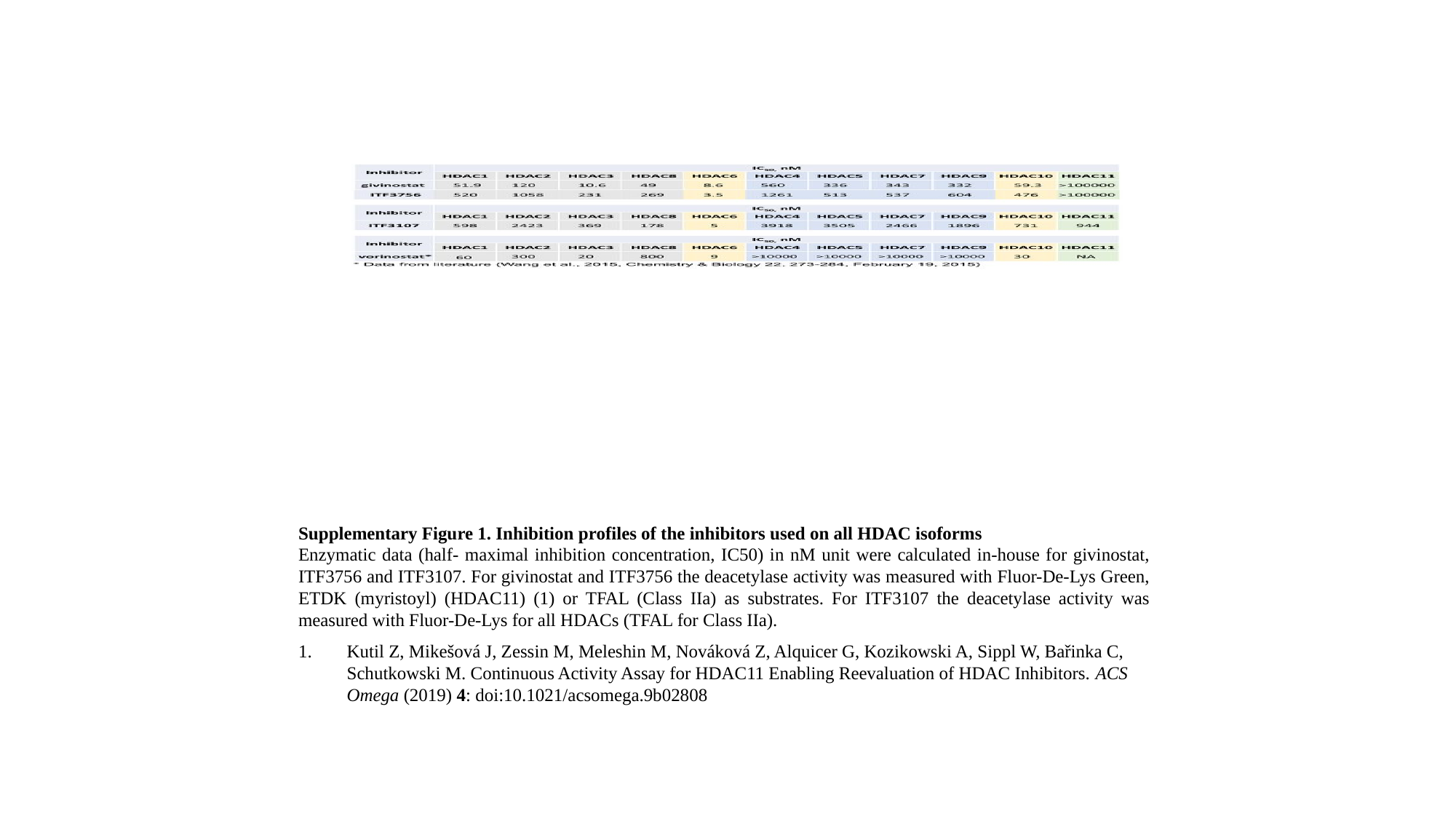

Supplementary Figure 1. Inhibition profiles of the inhibitors used on all HDAC isoforms
Enzymatic data (half- maximal inhibition concentration, IC50) in nM unit were calculated in-house for givinostat, ITF3756 and ITF3107. For givinostat and ITF3756 the deacetylase activity was measured with Fluor-De-Lys Green, ETDK (myristoyl) (HDAC11) (1) or TFAL (Class IIa) as substrates. For ITF3107 the deacetylase activity was measured with Fluor-De-Lys for all HDACs (TFAL for Class IIa).
1. 	Kutil Z, Mikešová J, Zessin M, Meleshin M, Nováková Z, Alquicer G, Kozikowski A, Sippl W, Bařinka C, Schutkowski M. Continuous Activity Assay for HDAC11 Enabling Reevaluation of HDAC Inhibitors. ACS Omega (2019) 4: doi:10.1021/acsomega.9b02808

## Slide 2
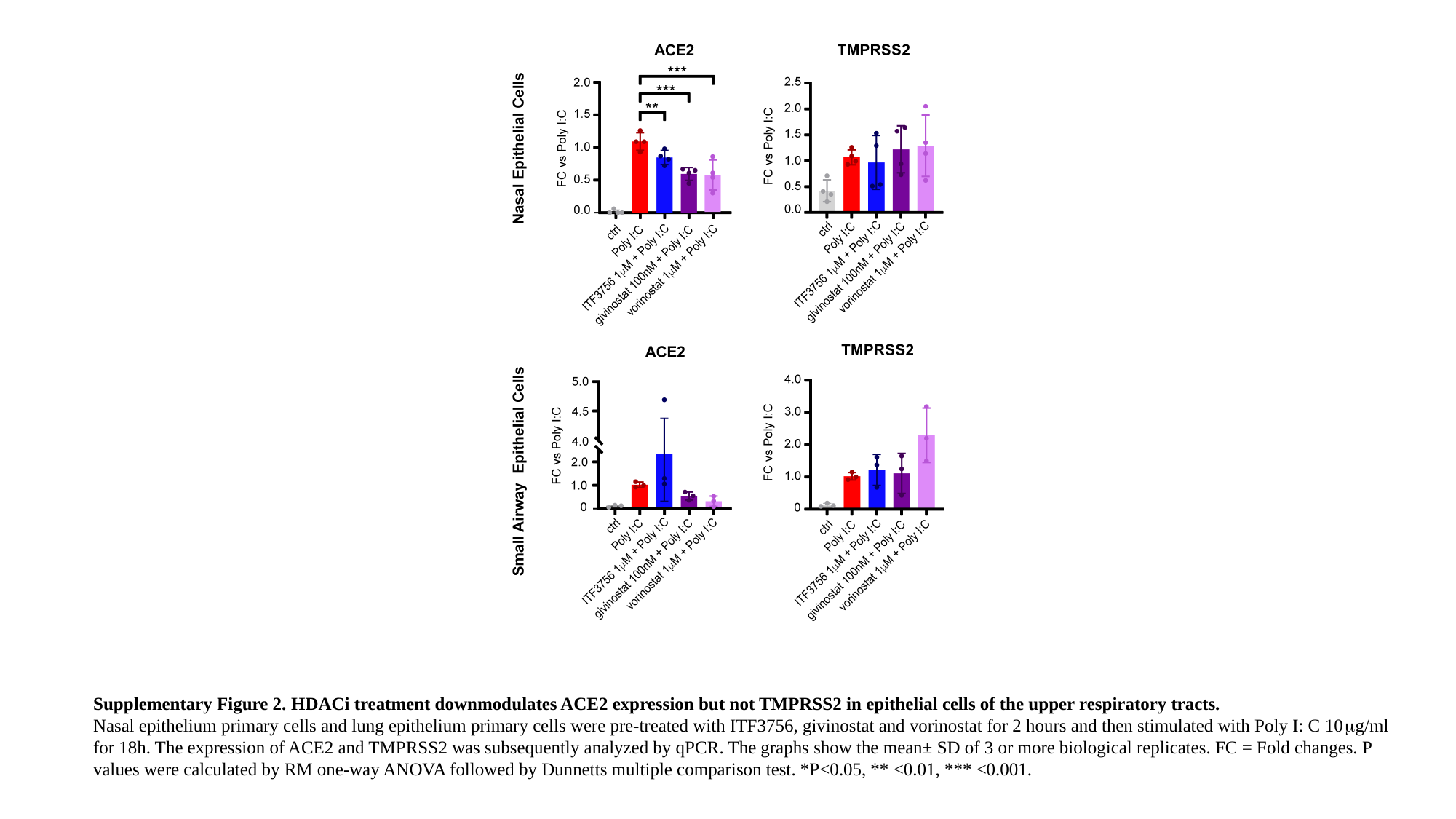

Supplementary Figure 2. HDACi treatment downmodulates ACE2 expression but not TMPRSS2 in epithelial cells of the upper respiratory tracts.
Nasal epithelium primary cells and lung epithelium primary cells were pre-treated with ITF3756, givinostat and vorinostat for 2 hours and then stimulated with Poly I: C 10μg/ml for 18h. The expression of ACE2 and TMPRSS2 was subsequently analyzed by qPCR. The graphs show the mean± SD of 3 or more biological replicates. FC = Fold changes. P values were calculated by RM one-way ANOVA followed by Dunnetts multiple comparison test. *P<0.05, ** <0.01, *** <0.001.

## Slide 3
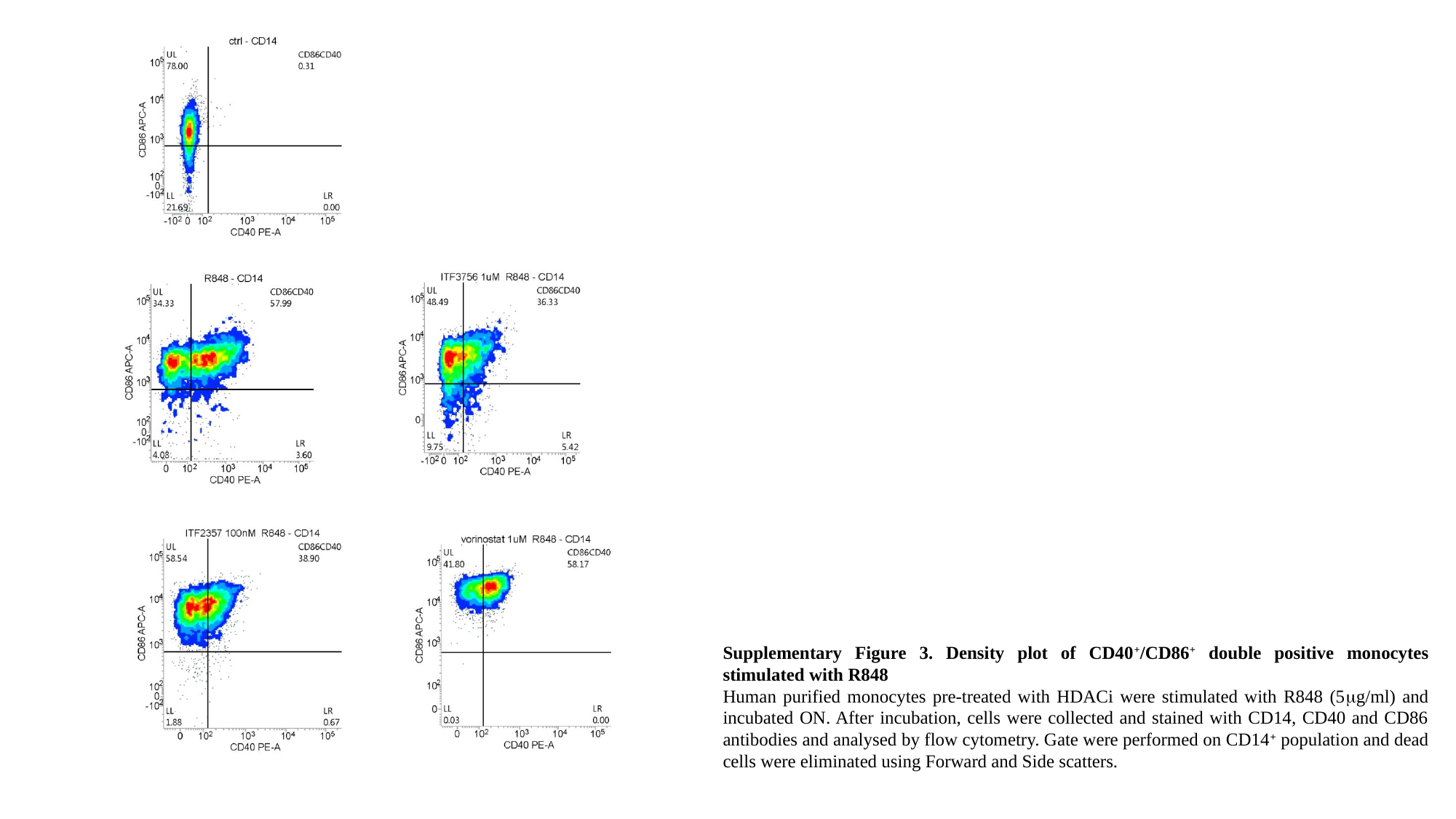

Supplementary Figure 3. Density plot of CD40+/CD86+ double positive monocytes stimulated with R848
Human purified monocytes pre-treated with HDACi were stimulated with R848 (5mg/ml) and incubated ON. After incubation, cells were collected and stained with CD14, CD40 and CD86 antibodies and analysed by flow cytometry. Gate were performed on CD14+ population and dead cells were eliminated using Forward and Side scatters.

## Slide 4
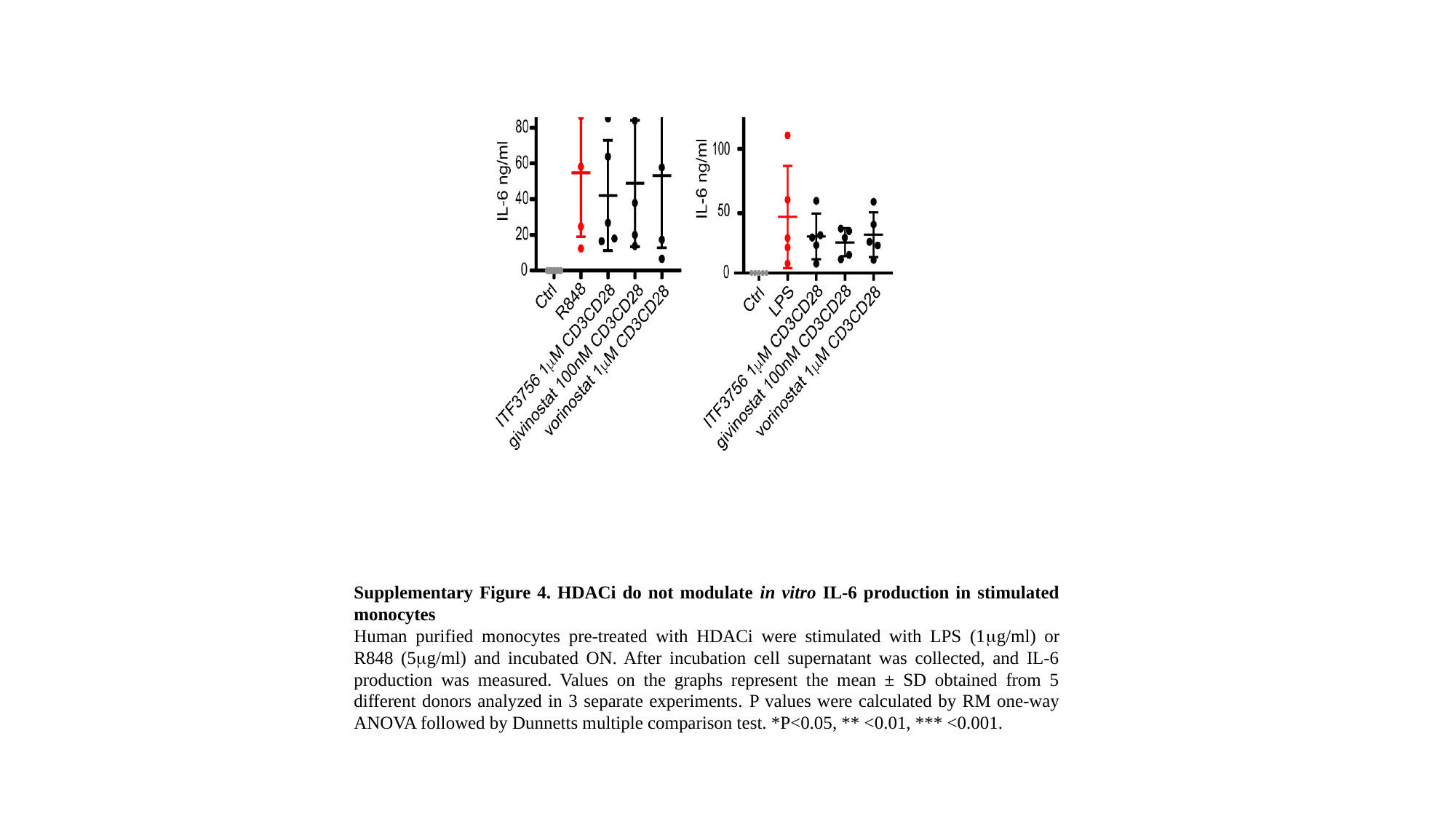

Supplementary Figure 4. HDACi do not modulate in vitro IL-6 production in stimulated monocytes
Human purified monocytes pre-treated with HDACi were stimulated with LPS (1mg/ml) or R848 (5mg/ml) and incubated ON. After incubation cell supernatant was collected, and IL-6 production was measured. Values on the graphs represent the mean ± SD obtained from 5 different donors analyzed in 3 separate experiments. P values were calculated by RM one-way ANOVA followed by Dunnetts multiple comparison test. *P<0.05, ** <0.01, *** <0.001.

## Slide 5
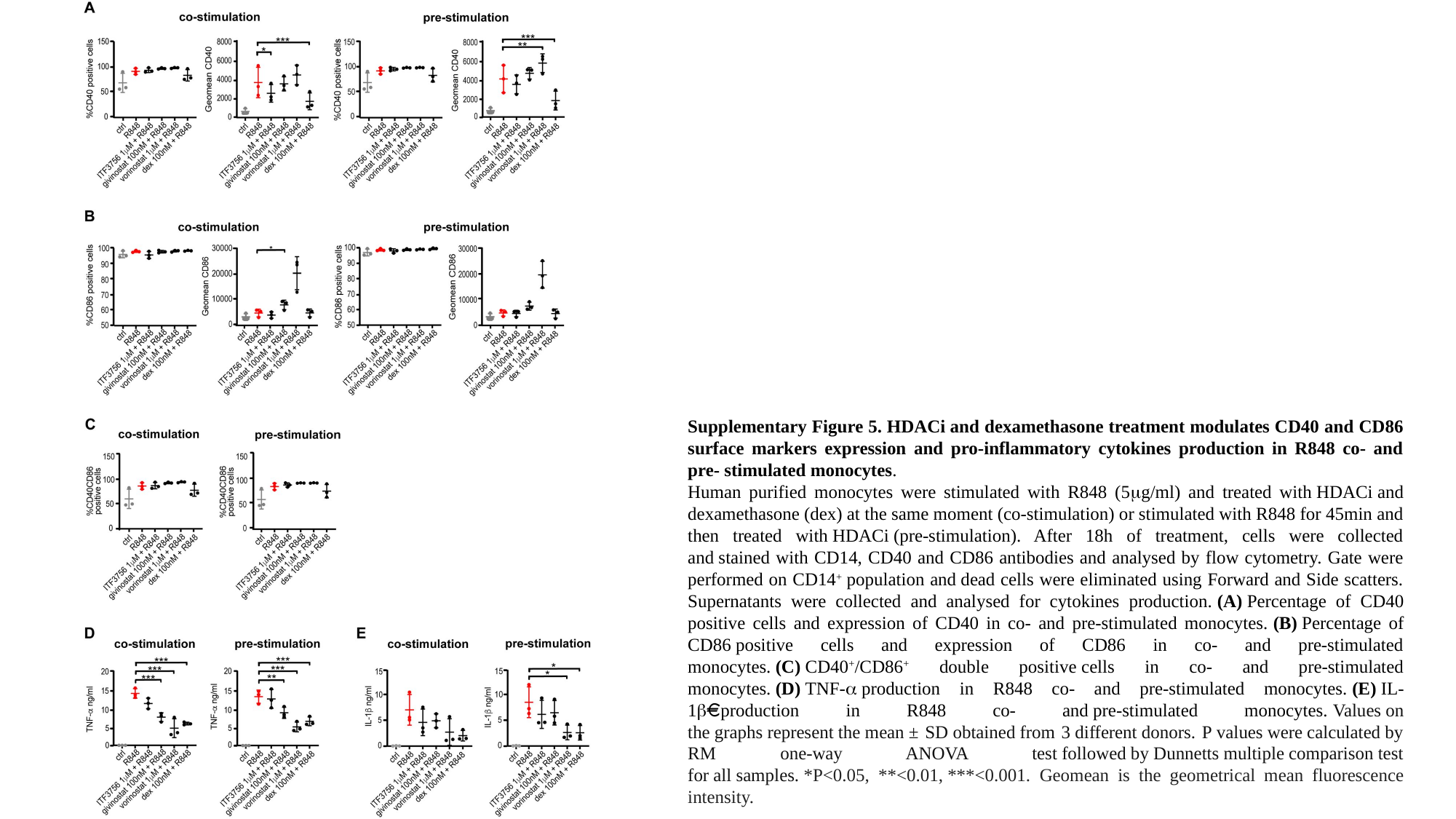

Supplementary Figure 5. HDACi and dexamethasone treatment modulates CD40 and CD86 surface markers expression and pro-inflammatory cytokines production in R848 co- and pre- stimulated monocytes.
Human purified monocytes were stimulated with R848 (5μg/ml) and treated with HDACi and dexamethasone (dex) at the same moment (co-stimulation) or stimulated with R848 for 45min and then treated with HDACi (pre-stimulation). After 18h of treatment, cells were collected and stained with CD14, CD40 and CD86 antibodies and analysed by flow cytometry. Gate were performed on CD14+ population and dead cells were eliminated using Forward and Side scatters. Supernatants were collected and analysed for cytokines production. (A) Percentage of CD40 positive cells and expression of CD40 in co- and pre-stimulated monocytes. (B) Percentage of CD86 positive cells and expression of CD86 in co- and pre-stimulated monocytes. (C) CD40+/CD86+ double positive cells in co- and pre-stimulated monocytes. (D) TNF-a production in R848 co- and pre-stimulated monocytes. (E) IL-1b production in R848 co- and pre-stimulated monocytes. Values on the graphs represent the mean ± SD obtained from 3 different donors. P values were calculated by RM one-way ANOVA test followed by Dunnetts multiple comparison test for all samples. *P<0.05, **<0.01, ***<0.001. Geomean is the geometrical mean fluorescence intensity.

## Slide 6
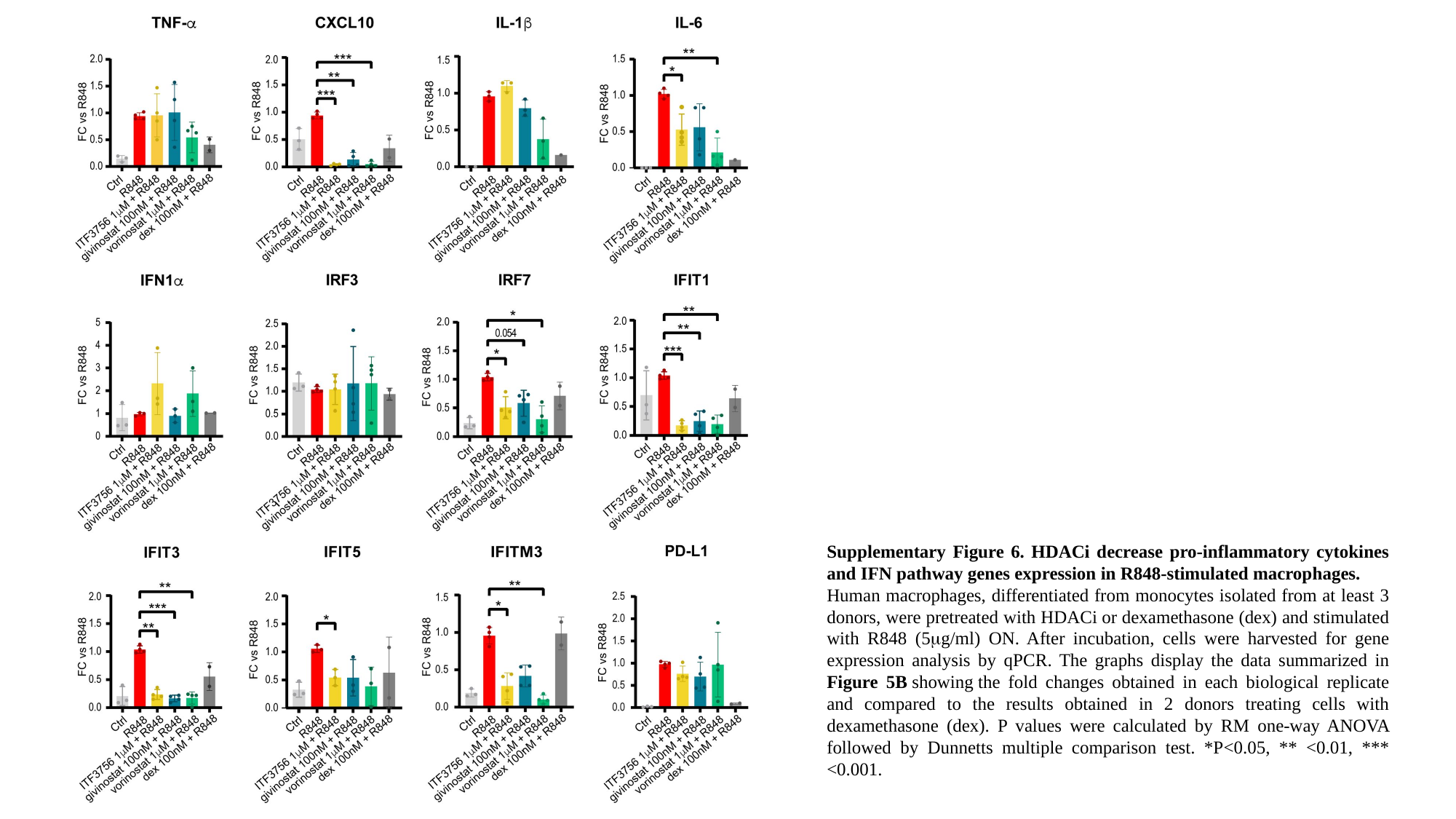

Supplementary Figure 6. HDACi decrease pro-inflammatory cytokines and IFN pathway genes expression in R848-stimulated macrophages.
Human macrophages, differentiated from monocytes isolated from at least 3 donors, were pretreated with HDACi or dexamethasone (dex) and stimulated with R848 (5mg/ml) ON. After incubation, cells were harvested for gene expression analysis by qPCR. The graphs display the data summarized in Figure 5B showing the fold changes obtained in each biological replicate and compared to the results obtained in 2 donors treating cells with dexamethasone (dex). P values were calculated by RM one-way ANOVA followed by Dunnetts multiple comparison test. *P<0.05, ** <0.01, *** <0.001.

## Slide 7
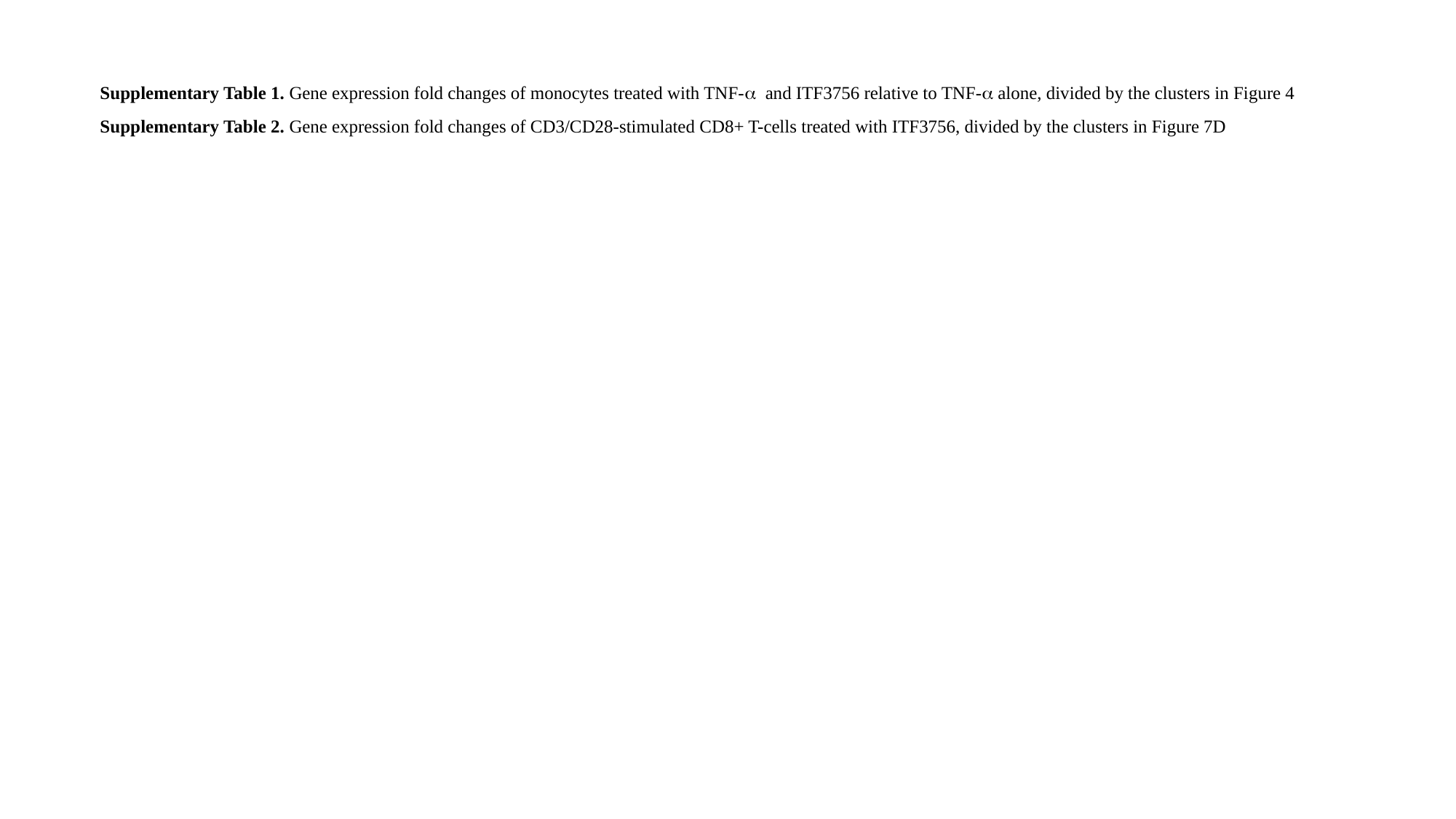

Supplementary Table 1. Gene expression fold changes of monocytes treated with TNF-a and ITF3756 relative to TNF-a alone, divided by the clusters in Figure 4
Supplementary Table 2. Gene expression fold changes of CD3/CD28-stimulated CD8+ T-cells treated with ITF3756, divided by the clusters in Figure 7D
